# Susceptibility gene identification and risk evaluation model construction by transcriptome-wide association analysis for salt sensitivity of blood pressure

**DOI:** 10.1186/s12864-024-10409-9

**Published:** 2024-06-18

**Authors:** Han Qi, Yun-Yi Xie, Xiao-Jun Yang, Juan Xia, Kuo Liu, Feng-Xu Zhang, Wen-Juan Peng, Fu-Yuan Wen, Bing-Xiao Li, Bo-Wen Zhang, Xin-Yue Yao, Bo-Ya Li, Hong-Dao Meng, Zu-Min Shi, Yang Wang, Ling Zhang

**Affiliations:** 1 Department of Epidemiology and Health Statistics, School of Public Health, Beijing Municipal Key Laboratory of Clinical Epidemiology, Capital Medical University, No.10 Youanmenwai, Beijing, 100069 China; 2 Beijing Key Laboratory of Mental Disorders, Beijing Anding Hospital, National Clinical Research Center for Mental Disorders & National Center for Mental Disorders, Capital Medical University, Beijing, 100088 China; 3 Faculty of Information Technology, Beijing University of Technology, Beijing, 100124 China; 4 Human Nutrition Department, College of Health Sciences, QU Health, Qatar University, Doha, Qatar; 5 Department of Cardiovascular Medicine, First Affiliated Hospital of Xi’an Jiaotong University, Xi’an, 710061 China

**Keywords:** Salt sensitivity of blood pressure, Transcriptome-wide association study, Polygenetic risk scores, Polygenic transcriptome risk scores, EpiSS study

## Abstract

**Background:**

Salt sensitivity of blood pressure (SSBP) is an intermediate phenotype of hypertension and is a predictor of long-term cardiovascular events and death. However, the genetic structures of SSBP are uncertain, and it is difficult to precisely diagnose SSBP in population. So, we aimed to identify genes related to susceptibility to the SSBP, construct a risk evaluation model, and explore the potential functions of these genes.

**Methods and results:**

A genome-wide association study of the systemic epidemiology of salt sensitivity (EpiSS) cohort was performed to obtain summary statistics for SSBP. Then, we conducted a transcriptome-wide association study (TWAS) of 12 tissues using FUSION software to predict the genes associated with SSBP and verified the genes with an mRNA microarray. The potential roles of the genes were explored. Risk evaluation models of SSBP were constructed based on the serial *P *value thresholds of polygenetic risk scores (PRSs), polygenic transcriptome risk scores (PTRSs) and their combinations of the identified genes and genetic variants from the TWAS. The TWAS revealed that 2605 genes were significantly associated with SSBP. Among these genes, 69 were differentially expressed according to the microarray analysis. The functional analysis showed that the genes identified in the TWAS were enriched in metabolic process pathways. The PRSs were correlated with PTRSs in the heart atrial appendage, adrenal gland, EBV-transformed lymphocytes, pituitary, artery coronary, artery tibial and whole blood. Multiple logistic regression models revealed that a PRS of *P* < 0.05 had the best predictive ability compared with other PRSs and PTRSs. The combinations of PRSs and PTRSs did not significantly increase the prediction accuracy of SSBP in the training and validation datasets.

**Conclusions:**

Several known and novel susceptibility genes for SSBP were identified via multitissue TWAS analysis. The risk evaluation model constructed with the PRS of susceptibility genes showed better diagnostic performance than the transcript levels, which could be applied to screen for SSBP high-risk individuals.

**Supplementary Information:**

The online version contains supplementary material available at 10.1186/s12864-024-10409-9.

## Introduction

Salt sensitivity of blood pressure (SSBP) is an intermediate phenotype of hypertension and is recognized as a parallel change in blood pressure following salt intake or salt depletion [1]. Individuals who manifest large blood pressure increases during salt intake or decreases in response to salt depletion are categorized as “salt sensitive (SS)”, and others are “salt resistant (SR)” [2]. SSBP is a predictor of long-term cardiovascular events and death [3–5]. Individuals with SSBP exhibit a non-dipper blood pressure pattern, which could increase the variability of blood pressure and is therefore associated with increased risks of target organ damage [6]. Early identification of SSBP is highly important for improving the prognosis of patients with hypertension and preventing long-term cardiovascular events.

Genetically, the heritability of SSBP is approximately 74% and 50% for black and Chinese individuals, respectively, suggesting that genetic factors play important roles in the pathogenesis of SSBP [7]. Genome-wide association studies (GWASs) seem to be a milestone in discovering the genetic associations of SSBP. The GenSalt study published GWAS findings on blood pressure sodium sensitivity and identified several novel loci [8]. A polygenic approach is needed to integrate the effects of individual variants to improve their predictive value. The polygenic risk score (PRS) is a prominent approach for grouping the effects of multiple loci and measuring the genetic risks of complex diseases effectively [9]. The PRS can be used for population risk stratification, treatment selection and prognosis estimation. To date, one PRS based on 42 known variants for SSBP has been published, and the results showed that PRS was significantly associated with SSBP [10]. The effects of novel loci on the genetic associations of SSBP are still uncertain.

In recent decades, researchers have found obvious limitations for GWASs because many GWAS-identified variants are located in noncoding regions. It is difficult to fully elucidate the functions of these variants and the genetic structure of complex diseases based on GWAS [11]. Some novel approaches have been developed, such as the transcriptome-wide association study (TWAS), which can integrate GWAS and expression quantitative trait loci (eQTLs) and has been widely used in identifying risk genes for complex diseases, such as neuropsychiatric diseases [12, 13], cancer [14] and cardiovascular diseases [15, 16]. TWAS can help to detect candidate genes for complex diseases even with a relatively small set of reference panels and lower multiple-testing burdens [17, 18]. As a systematic disease, multiple tissues are involved in the pathogenesis of systemic SSBP, such as renal, arterial, heart, whole blood and other cardiovascular-related tissues [19]. SSBP susceptibility genes and the best eQTLs can be accurately identified from multiple tissues using the TWAS. Furthermore, polygenic transcriptome risk scores (PTRSs), which are based on transcript levels rather than genetic variants, have better portability across populations and may complement PRSs in predicting genetic risks for complex diseases [20]. The combination of PRSs with PTRSs may improve the predictive value of traits [21].

**Fig. 1:**
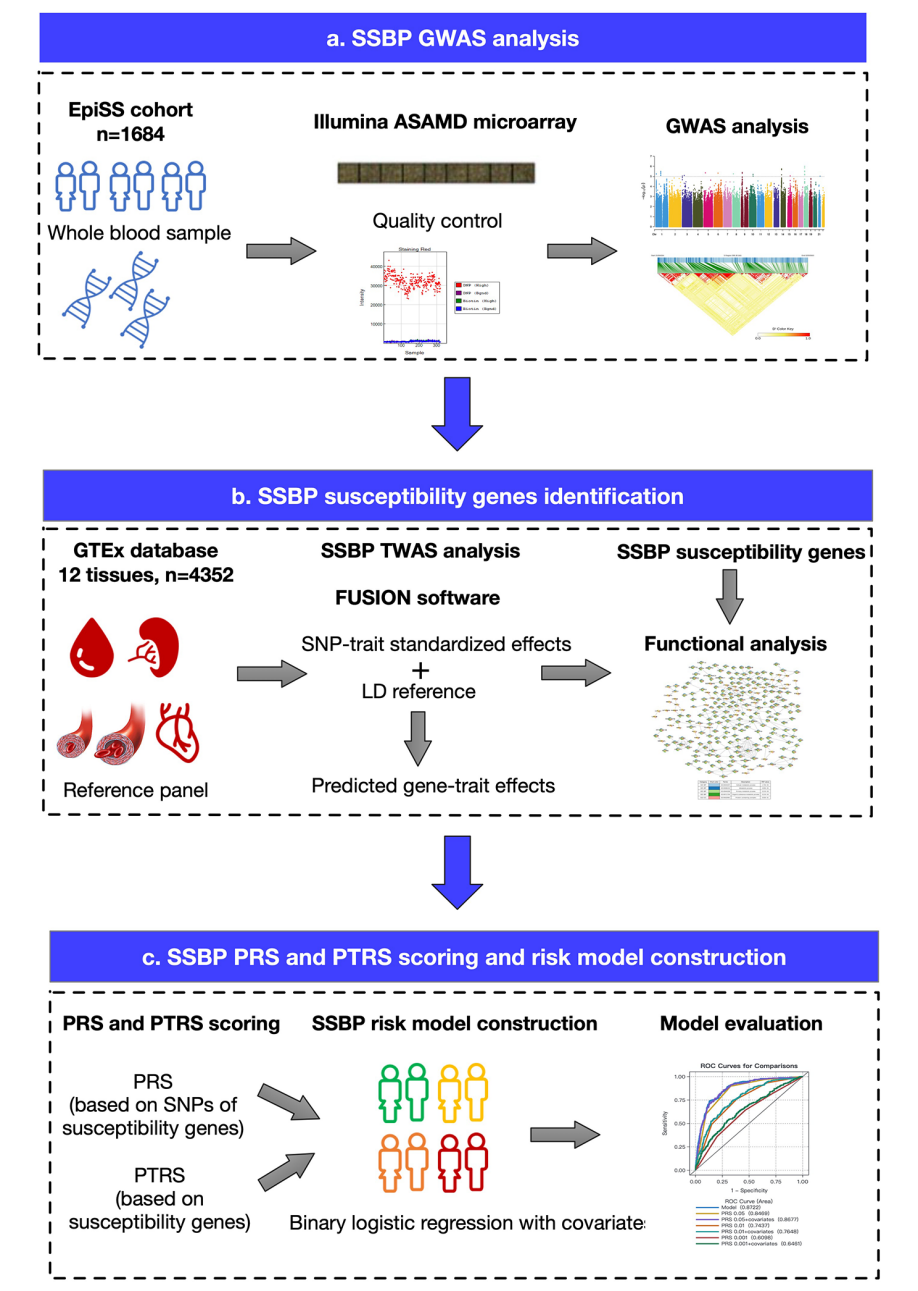
Study design flowchart. **a**, The Illumina ASAMD microarray was used for the EpiSS cohort genotyping, and GWAS was conducted to explore the SNPs associated with SSBP. **b**, The reference panel of 12 tissues from the GTEx v8 database was downloaded, and TWAS analysis was applied to identify the SSBP susceptibility genes using FUSION software. The functions of the susceptibility genes were preliminarily explored. c, The PRS and PTRS were calculated based on the TWAS results, and SSBP risk models were constructed to evaluate the risk of SSBP in the population

In this study, we aimed to (1) perform a tissue-specific TWAS analysis to identify the susceptibility genes of SSBP based on GWAS data; (2) validate the associations of the genes with SSBP; (3) explore the potential functions of the target genes; and (4) use the PRS and PTRS calculated from the susceptibility genes and genetic variants to construct risk evaluation models of SSBP and compare the performance. The overall flowchart was shown in Fig. 1.

## Materials and methods

### Participants

Participants were recruited from the systemic epidemiology of salt sensitivity (EpiSS) cohort study. The details of the protocol were described elsewhere [22]. Briefly, participants who were 35–70 years old and lived in 11 study centres in Beijing and Liaoning provinces were invited to participate in EpiSS. In the EpiSS, the salt sensitivity of blood pressure was identified using the modified Sullivan’s acute oral saline load and diuresis shrinkage test (MSAOSL-DST). This test required participants to take 1,000 mL of 0.9% saline orally for 30 min. Blood pressure was measured at baseline (time 1), 2 h after sodium loading (time 2), and 2 h after diuresis reduction (time 3). Participants were divided into SS or SR individuals according to the change in three mean arterial pressures (MAP_1 − 3_). Participants with MAP_2_-MAP_1_ ≥ 5 mmHg or MAP_3_-MAP_2_≤-10 mmHg were defined as having SS, and the others were defined as having SR [23]. Urine and peripheral blood samples were collected during the questionnaire and physical examinations. This study was approved by the Ethics Committee of Capital Medical University (no. Z2023SY025) and was registered with the WHO Clinical Trials Registry Platform (ChiCTR-EOC-16009980, November 23, 2016).

### DNA extraction, genotyping, and genome-wide association analysis

The DNA samples were first extracted and quantified using a Magnetic Bead Whole Blood Genomic DNA Extraction Kit (BioTeke, Beijing, China) and a Nanodrop 2000 spectrophotometer (Thermo Fisher Scientific, Waltham, MA, USA) with a standard operating process. Next, the gDNA samples from 2057 participants were genotyped using the Illumina Infinium Asian Screening Array BeadChip-24 v1.0 (ASA) (Illumina, San Diego, CA, USA). This chip contained 740,000 SNPs, including nearly 50,000 SNPs specially customized for the Chinese population with known susceptibility to multiple diseases [24]. SNPs with a call rate < 0.95, an MAF < 0.01, a Hardy‒Weinberg equilibrium < 1 × 10^− 4^, and SNPs on the sex chromosome were excluded. Similarly, samples with call rates < 0.95, abnormal heterozygosity rates > 6SD, and PI_HAT > 0.25 were excluded. In total, 1684 participants with 483,002 SNPs were included. The genipe automated genome-wide imputation pipeline was used to impute, report and analyse the data [25]. After imputation, 4,241,225 SNPs were obtained for further analysis.

The association analysis between SNPs and SSBP was conducted using an additive genetic model with the condition of SSBP (SS or SR) as the dependent variable and SNPs as the independent variables adjusted for the covariates of age, sex, area, hypertension, fasting glucose (FBG), triglycerides (TG) and body mass index (BMI). The selection of covariates was based on the associations of these variables with SSBP in this study and previously published statements [1]. The association analysis and clumping of variants were conducted in Plink v1.09.

### Transcriptome-wide association analysis

The SNP and eQTL data of 12 tissues (adipose subcutaneous, adipose visceral, adrenal gland, artery aorta, artery coronary, artery tibial, blood-EBV-transformed lymphocytes, heart atrial appendage, heart-left ventricle, kidney cortex, pituitary, and whole blood) (*n* = 4,352) were retrieved from the GTEx v8.0 database (https://gtexportal.org/home/) to further identify the susceptibility genes associated with SSBP [26]. The TWAS analysis was conducted with FUSION software (http://gusevlab.org/projects/fusion). Five gene expression models were used to construct the prediction models, including the best linear unbiased predictor computed from all SNPs (blup), Bayesian sparse linear model (BSLMM), elastic-net regression (ENET), least absolute shrinkage and selection operator (LASSO), and single best eQTL (TOP1) models. The TWAS results with the best-performing prediction model were output. A series of *P* values (0.05, 0.01, 0.001, 0.0001) were used to identify the susceptibility genes. Furthermore, conditional analysis was performed to distinguish the independent associations of genes (*P* < 0.05) with SSBP from the genes that were not significant when accounting for the predictive expression of other genes in a given locus [18]. Manhattan plots of the TWAS analysis results were generated using the “CMplot” package in R software.

### Validation of potential genes

The potential genes associated with SSBP were further validated using the Agilent SBC human (4*180 K) ceRNA array v1.0. The details of the experimental method were described elsewhere [27]. The “limma” package of R software was used to identify the differently expressed genes (DEGs) between SS and SR (*n* = 20). The microarray data were submitted to the Gene Expression Omnibus (GEO) database (GSE135111).

### PRS and PTRS scoring

The PRS scoring method was used to calculate the genetic risk of individuals based on TWAS analysis. The most significant GWAS SNPs in the locus and the best eQTL in the locus of TWAS analysis were extracted to calculate the PRS predictive values. The genotypes of the SNPs in susceptible genes were derived from the “ped” document. Then, the PRSs were calculated by multiplying the number of risk alleles and the effect size of each SNP with the SSBP (ln odds ratios) in each subset of susceptible genes with the series of P thresholds. The formula was as follows [28]:

$${PRS}_{PT,j}=\sum _{i=1}^{m}{\beta }_{i}{G}_{i,j}$$

*PT* = the series of *P* value thresholds (0.05, 0.01, 0.001, 0.0001).

*I* = the number of SNPs under the thresholds.

*β* = the effect size of SNPs (ln odds ratios for categorical phenotype).

*G* = the genotype of SNPs (0, 1, 2).

Similarly, the PTRSs were calculated with TWAS *P* thresholds of 0.0001, 0.001, 0.01, and 0.05. Specifically, the PTRS was also calculated for the DEGs that overlapped between the TWAS and the gene microarray. The individual-level data predicted using 12 tissue-specific weight files and packages were used to generate aggregate and tissue-specific individual-level data. The effect sizes estimated in the FUSION associations are the PTRS weights. The formula for PTRS was as follows [20]:

$${PTRS}_{PT,j}=\sum _{i=1}^{m}{\beta }_{i}{T}_{i,j}$$

*PT* = the series of *P* value thresholds (0.05, 0.01, 0.001, 0.0001).

*I* = the number of genes identified by the TWAS under the thresholds.

*β* = the effect size of genes (TWAS. Z value).

*T* = the predicted individual-level gene expression data.

Furthermore, the PRS and PTRS were combined using the weights calculated from the principal component analysis (PCA). First, the correlation matrix between different *P* thresholds of PRS and PTRS was constructed to select the potential combination of PRSs and PTRSs with stronger correlations. Then, the PCAs were performed to determine the weights (c_1_ and c_2_) for the PRS and PTRS combination. The calculations of c_1_ and c_2_ were the normalization of the weighted average of the coefficients of the variables in the linear combination of the PCA with the variance contribution rate as weights.

### Functional exploration analysis

The functions of SSBP susceptibility genes (*P* < 0.05 according to the TWAS analysis) were preliminarily explored using a protein‒protein interaction (PPI) network, GO enrichment analysis, Kyoto Encyclopaedia of Genes and Genomes (KEGG) pathway analysis and cell type-specific enrichment analysis. STRING 11.5 in Cytoscape 3.9.1 software was used to conduct the network analysis and visualize the susceptibility gene network [29]. The genes that were not connected in the network were removed. The top 5 enrichment analysis results from the GO and KEGG pathway analyses are shown with different colours in the plot. Additionally, the cell types in which the susceptibility genes were enriched were explored using web-based cell type-specific enrichment analysis of genes (WebCSEA) [30]. The top 20 enriched cell types and tissues were visualized in a scatter plot.

### Statistical analysis

SAS 9.4 software (SAS Institute, Cary, NC, US) was used to conduct the data analysis. The baseline characteristics of continuous variables are described as the mean ± standard deviation (normal distribution) or median and interquartile range (IQR) (skewed distribution). The categorical variables are described as the number of patients and percentage. Two-way independent sample t tests or Mann‒Whitney U tests were used to compare the differences in continuous variables between the SS and SR groups. The chi-square test or Fisher’s exact test was used for the comparison of categorical variables between the SS and SR groups.

The whole dataset was randomly divided into a training dataset (70%) and a validation dataset (30%). A binary logistic regression model was used to explore the associations between SSBP and PRS or PTRS after adjusting for covariates. The odds ratio (OR) and 95% CIs were calculated. The dependent variable was the status of SSBP (SS or SR). The PRS, PTRS and their combinations with different *P* thresholds were separately input into the models as independent variables. The PRS and PTRS were categorized into Q1-Q4 according to the 25th, 50th and 75th percentiles, respectively. Considering the potential collinearity between FBG and a history of diabetes and between MAP_1_, TG, and LDL-C and a history of hypertension, we included a history of diabetes and hypertension in the model, which might be more stable than the results of a single examination. Age, sex, and BMI were also included in the multivariate models because they were influencing factors of SSBP. The area under the curve (AUC) of the receiver operating characteristic (ROC) curve was calculated to evaluate the performance of the models. Additionally, the 10-fold cross-validation was used to validate the model internally. A two-sided *P* value < 0.05 was considered to indicate statistical significance.

## Results

### Baseline characteristics of participants

Table 1 shows the baseline characteristics of the participants included in the GWAS between SS and SR. In total, 1684 participants were recruited from the EpiSS cohort study for GWAS. Among them, 1,199 (71.2%) were SR, and 485 (28.8%) were SS. The median age was 59.0 years, and 73.5% were women. Univariate analysis revealed that education, smoking status, history of diabetes, FBG, TG, LDL-C, MAP_1_ (mean arterial blood pressure at baseline) and MAP_2_ (mean arterial blood pressure 2 h after acute salt loading) were significantly different between the SS and SR groups (*P* < 0.05).

**Table 1:** Baseline characteristics of the participants

Variables	Total (*n* = 1,684)	SR (*n* = 1,199)	SS (*n* = 485)	*P* value
Sex, n (%)				0.082^a^
Women	1237 (73.5)	895 (74.6)	342 (70.5)	
Men	447 (26.5)	304 (25.4)	143 (29.5)	
Marriage, n (%)				0.119^b^
Unmarried	6 (0.4)	3 (0.3)	3 (0.7)	
Married	1436 (93.6)	1009 (93.2)	427 (94.5)	
Divorced	25 (1.6)	16 (1.5)	9 (2.0)	
Widowed	68 (4.4)	55 (5.1)	13 (2.9)	
Education, n (%)				0.021^a^
Postgraduate and above	56 (3.4)	33 (2.8)	23 (4.8)	
College and university	252 (15.2)	177 [15]	75 (15.8)	
High school	517(31.2)	355 (30.1)	162 (34.1)	
Middle school	648 (39.1)	485 (41.1)	163 (34.3)	
Primary school	166 (10.0)	122 (10.3)	44 (9.3)	
Illiteracy	17 (1.0)	9 (0.8)	8 (1.7)	
Smoking, n (%)				0.005^a^
No	1426 (84.7)	1034 (86.2)	392 (80.8)	
Yes	258 (15.3)	165 (13.8)	93 (19.2)	
Drinking Frequency, n (%)				0.427^a^
Almost everyday	484 (28.9)	340 (28.5)	144 (29.8)	
3–5 times/week	96 (5.7)	62 (5.2)	34 (7.0)	
1-3times/week	66 (3.9)	44 (3.7)	22 (4.6)	
1-3times/month	132 (7.9)	94 (7.9)	38 (7.9)	
Never	896 (53.5)	651 (54.7)	245 (50.7)	
Hypertension, n (%)				0.931^a^
No	834 (49.5)	593 (49.5)	241 (49.7)	
Yes	850 (50.5)	606 (50.5)	244 (50.3)	
Diabetes, n (%)				0.037^a^
No	1414 (84.0)	1021 (85.2)	393 (81.0)	
Yes	270 (16.0)	178 (14.8)	92 (19.0)	
Age (years), median (IQR)	59.0 (9.4)	59.0 (9.7)	59.0 (10.0)	0.635^c^
BMI (kg/m^2^), median (IQR)	25.9 (4.4)	25.9 (4.5)	25.9 (4.1)	0.990^c^
FBG (mmol/L), median (IQR)	5.4 (1.2)	5.4 (1.1)	5.6 (1.4)	0.010^c^
TC (mmol/L), median (IQR)	5.0 (1.4)	5.0 (1.4)	5.0 (1.3)	0.306^c^
TG (mmol/L), median (IQR)	1.6 (1.4)	1.7 (1.3)	1.5 (1.3)	0.036^c^
LDL-C (mmol/L), median (IQR)	2.3 (1.4)	2.3 (1.4)	2.1 (1.3)	0.011^c^
HDL-C (mmol/L), median (IQR)	1.4 (1.3)	1.4 (1.2)	1.4 (1.5)	0.602^c^
MAP_1_ (mmHg), mean ± SD	97.3 ± 12.0	98.6 ± 11.8	94.0 ± 11.8	< 0.001^d^
MAP_2_ (mmHg), median (IQR)	91.8 (16.5)	89.8 (16.0)	96.3 (15.4)	< 0.001^c^
MAP_3_ (mmHg), median (IQR)	92.3 (16.3)	92.5 (16.3)	91.5 (16.7)	0.687^c^

Note: a, chi-square test; b, Fisher’s exact test; c, Mann‒Whitney U test; d, two independent sample t test; SS, salt sensitivity; SR, salt resistance; BMI, body mass index; FBG, fasting blood glucose; TC, total cholesterol; TG, triglyceride; LDL-C, low density lipoprotein cholesterol; HDL-C, high density lipoprotein cholesterol; MAP_1_, mean arterial blood pressure at baseline; MAP_2_, mean arterial blood pressure 2 h after acute salt loading; MAP_3_, mean arterial blood pressure 2 h after diuresis shrinkage

### GWAS analysis of SSBP

The GWAS analysis revealed that 36 SNPs were significantly and genome-wide associated with SSBP (*P* < 1 × 10^−5^). A summary of the results for the 36 SNPs was provided in Supplementary Table 1. The SNPs were intron variants and located on chromosomes 1, 3, 5. 6, 8, 9, 10, 14, 15, 18 and 21 (Supplementary Fig. 1). The chromosome 18 had the largest number of SNPs. Among the 36 SNPs, 26 were positively associated with SSBP, and rs143884031 had the strongest correlation with SSBP with an OR of 3.572 (95% CI: 2.062–6.188, *P* = 5.57 × 10^− 6^). Ten SNPs were negatively associated with SSBP, among which rs138139129 had the strongest correlation with SSBP, with an OR of 0.286 (95% CI: 0.165–0.498, *P* = 9.43 × 10^− 6^). The power of the one-stage GWAS analysis of SSBP was calculated using the Genetic Association Study (GAS) Power Calculator (http://csg.sph.umich.edu/abecasis/cats/gas_power_calculator/index.html). We inputted the number of cases (n = 485), number of controls (n = 1,199), significant level (1 × 10^−5^), disease allele frequency (rs1904694, 0.358), prevalence of SSBP (0.35) and genotype relative risk (rs1904694, 1.58) into the calculator and found that the statistical power was 0.996 [31].

### TWAS analysis of SSBP

**Fig. 2:**
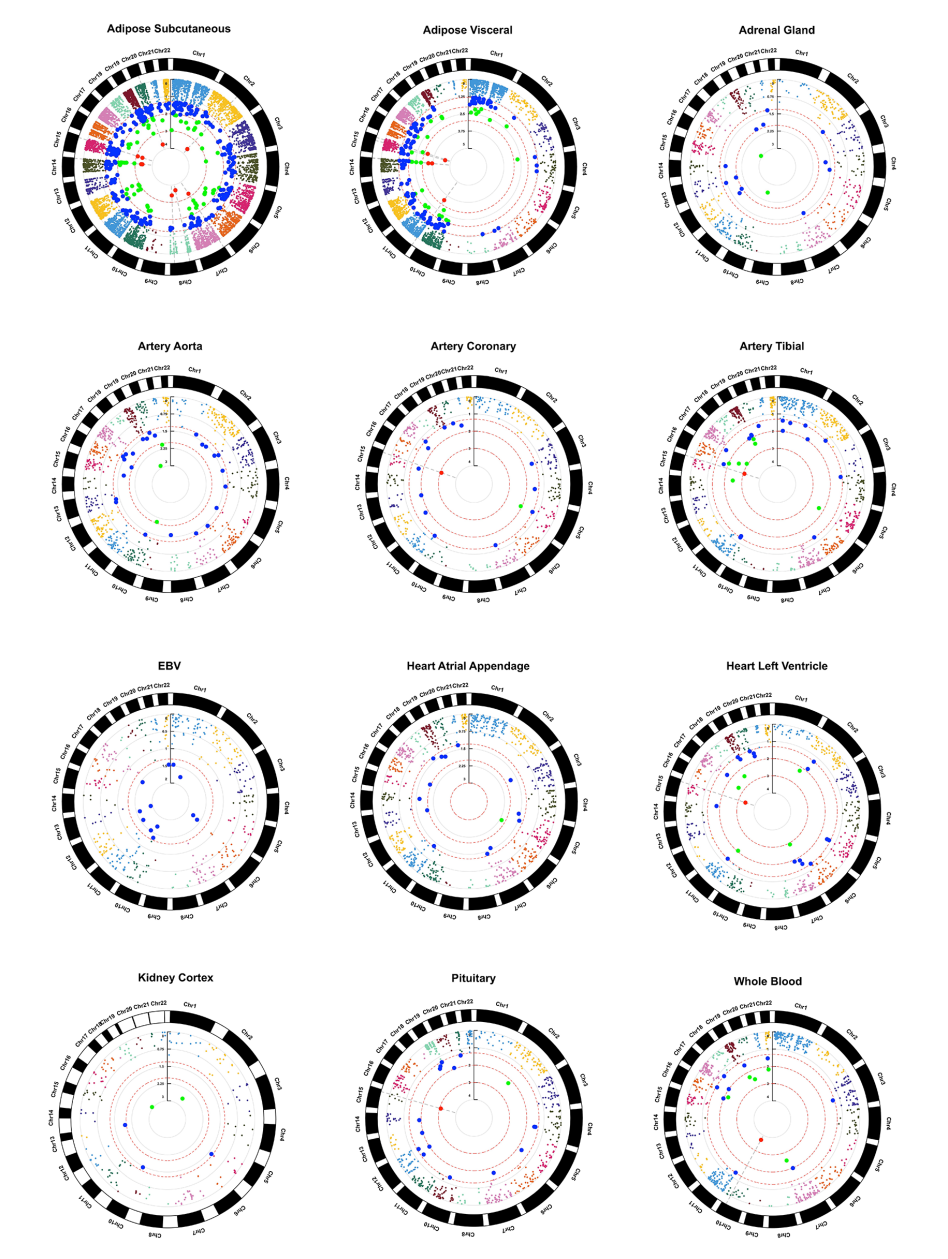
Circle Manhattan plots of the association results from the SSBP TWAS of 12 tissues. Each point represents a single gene. The blue, green and red points represent TWASs with *P* < 0.05, < 0.01 and < 0.001, respectively

The summary results of GWAS were imported into FUSION software, and 18,028 genes were identified to be associated with SSBP. Among these genes, 2,605 had *P* < 0.05, 585 had *P* < 0.01, 66 had *P* < 0.001, and seven had *P* < 0.0001 (*GRAMD2A*, *PARP6*, *GRF2E2*, *CEP85*, *ENSG00000272630*, *SRRM4*, *UBXN11*). The distributions of these genes on the chromosomes could be found in the circle Manhattan plot in Fig. 2. Notably, four TWAS-identified genes were widely present in all 12 tissues (*EIF5A, H3-3 A, INTS1, and SEPTIN7P14*), six in 11 tissues and nine in 10 tissues. Conditional analysis was further conducted to identify the genes significantly associated with SSBP. The results showed that the GWAS signals of 20 genes remained significant after the associations of the functional genes in the locus were removed (Supplementary Table 2).

To further verify the associations between the susceptibility genes and SSBP, we validated the results of the TWAS analysis with an SSBP mRNA microarray. There were 18,853 mRNAs in the microarray. A total of 1373 mRNAs were significantly differentially expressed between SS and SR (*P* < 0.05). We found that 69 genes were overlapped between the TWAS analysis and microarray validation. The details of the 69 susceptibility genes were shown in Table 2. However, there were no overlapping genes between the TWAS conditional analysis and the susceptibility genes that were verified in the microarray.

**Table 2:** Information on the 69 differentially expressed susceptibility genes of SSBP identified by the TWAS

No	Gene Symbol	Chromosome	Start	End	Tissues	TWAS Z Value	TWAS *P* Value	Microarray *P* Value
1	*GDPGP1*	15	90233808	90245811	Adipose Subcutaneous	3.335	0.001	0.012
2	*ANKS1A*	6	34889255	35091406	Adipose Subcutaneous	2.973	0.003	0.048
3	*AFAP1L2*	10	114294824	114404756	Adipose Subcutaneous	2.905	0.004	0.002
4	*CYBRD1*	2	171522247	171558129	Adrenal Gland	2.795	0.005	0.001
5	*LIPN*	10	88759982	88779626	Cells EBV-transformed lymphocytes	-2.729	0.006	0.001
6	*ZSWIM7*	17	15976560	15999717	Whole Blood	2.688	0.007	0.002
7	*CHST11*	12	104455295	104762014	Adrenal Gland	-2.687	0.007	0.006
8	*COG4*	16	70480568	70523560	Heart Left Ventricle	2.682	0.007	0.042
9	*PXMP4*	20	33702758	33720319	Adipose Subcutaneous	2.672	0.008	0.001
10	*YIPF4*	2	32277904	32316594	Adipose Visceral Omentum	-2.670	0.008	0.001
11	*CPNE1*	20	35626031	35664956	Adipose Subcutaneous	-2.587	0.010	0.015
12	*CPSF2*	14	92121969	92172145	Adrenal Gland	2.569	0.010	0.045
13	*ZNF780A*	19	40090752	40090943	Adrenal Gland	2.524	0.012	0.006
14	*GRB10*	7	50590068	50793453	Artery Aorta	2.524	0.012	0.008
15	*ZNF773*	19	57499938	57518437	Artery Aorta	2.522	0.012	0.017
16	*ERCC6*	10	49434881	49539538	Pituitary	2.490	0.013	0.021
17	*ZNF701*	19	52570287	52600149	Artery Tibial	2.468	0.014	0.035
18	*HPR*	16	72063226	72077246	Adipose Visceral Omentum	2.457	0.014	0.028
19	*TGFB1*	19	41330323	41353922	Adipose Visceral Omentum	2.453	0.014	0.045
20	*VN1R1*	19	57454790	57457140	Adrenal Gland	2.450	0.014	0.033
21	*AKAP13*	15	85380603	85749358	Adipose Subcutaneous	-2.436	0.015	0.006
22	*FBXL18*	7	5454425	5513809	Heart Left Ventricle	2.424	0.015	0.002
23	*EPC2*	2	148644751	148787569	Whole Blood	2.410	0.016	0.009
24	*CYP11B1*	8	142872357	142879825	Adrenal Gland	-2.396	0.017	0.024
25	*PLCB2*	15	40284256	40307935	Whole Blood	-2.328	0.020	0.041
26	*EIF3C*	16	28688558	28735730	Whole Blood	-2.326	0.020	0.011
27	*KCNQ2*	20	63400208	63472655	Pituitary	2.319	0.020	0.044
28	*CYP2U1*	4	107931549	107953461	Adipose Subcutaneous	-2.316	0.021	0.027
29	*NLRC3*	16	3539033	3577403	Kidney Cortex	-2.292	0.022	0.004
30	*GEMIN5*	5	154887411	154938211	Adipose Visceral Omentum	2.286	0.022	0.034
31	*CEP72*	5	612340	676616	Artery Coronary	-2.239	0.025	0.004
32	*PLCG2*	16	81779291	81962685	Heart Atrial Appendage	2.239	0.025	0.025
33	*HHIPL1*	14	99604538	99680569	Artery Aorta	-2.233	0.026	0.047
34	*TRAF3*	14	102777449	102911500	Cells EBV-transformed lymphocytes	-2.226	0.026	0.002
35	*EIF2B5*	3	184135358	184145311	Artery Tibial	2.220	0.026	0.032
36	*CD84*	1	160541098	160579496	Adipose Subcutaneous	2.213	0.027	0.006
37	*SNRNP35*	12	123458139	123473154	Whole Blood	-2.193	0.028	0.009
38	*ESRRB*	14	76310777	76501837	Adrenal Gland	2.192	0.028	0.049
39	*COX15*	10	99694293	99732127	Adipose Visceral Omentum	2.157	0.031	0.010
40	*MED4*	13	48075724	48095104	Adrenal Gland	2.152	0.031	0.022
41	*GSAP*	7	77310751	77416630	Adipose Subcutaneous	2.143	0.032	0.043
42	*USP37*	2	218568360	218568361	Heart Atrial Appendage	2.119	0.034	0.005
43	*ACSS1*	20	25006237	25058139	Heart Atrial Appendage	-2.109	0.035	0.049
44	*GTF2H1*	11	18322567	18367045	Artery Aorta	2.109	0.035	0.005
45	*SPHK2*	19	48624132	48624133	Adipose Subcutaneous	-2.107	0.035	0.030
46	*ANAPC16*	10	72216012	72235860	Artery Aorta	2.102	0.036	0.034
47	*DYNC1I2*	2	171687469	171750158	Heart Left Ventricle	-2.099	0.036	0.018
48	*DENND4B*	1	153929501	153946894	Pituitary	-2.096	0.036	0.001
49	*GP9*	3	129054845	129062406	Adipose Visceral Omentum	-2.096	0.036	0.043
50	*EPG5*	18	45800581	45967329	Artery Tibial	-2.088	0.037	0.006
51	*RAB40C*	16	589357	629268	Whole Blood	-2.080	0.037	0.017
52	*MMS19*	10	97458324	97498794	Artery Tibial	2.080	0.038	0.025
53	*E2F1*	20	33675477	33686385	Pituitary	2.069	0.039	0.001
54	*C4orf3*	4	119296419	119304445	Artery Aorta	-2.064	0.039	0.013
55	*MAT2A*	2	85539168	85545281	Artery Tibial	-2.059	0.039	0.030
56	*C14orf119*	14	23095505	23100456	Whole Blood	-2.050	0.040	0.014
57	*GTF2I*	7	74657718	74760692	Adipose Subcutaneous	-2.046	0.041	0.017
58	*PGM2L1*	11	74330316	74398433	Artery Aorta	2.033	0.042	0.012
59	*APMAP*	20	24962925	24992751	Artery Aorta	2.014	0.044	0.017
60	*FANCM*	14	45135930	45200890	Adipose Subcutaneous	-2.004	0.045	0.038
61	*GATAD1*	7	92447482	92495769	Adrenal Gland	2.003	0.045	0.003
62	*PGM1*	1	63593411	63660245	Adipose Subcutaneous	-2.003	0.045	0.032
63	*GIMAP4*	7	150567390	150573953	Cells EBV-transformed lymphocytes	-1.998	0.046	0.013
64	*ATXN3*	14	92058552	92106582	Heart Left Ventricle	1.994	0.046	0.003
65	*ARRB1*	11	75260122	75351661	Adipose Subcutaneous	1.981	0.048	0.033
66	*ALKBH8*	11	107502727	107565735	Adipose Visceral Omentum	1.978	0.048	0.023
67	*TTF2*	1	117060326	117107453	Adipose Subcutaneous	-1.972	0.049	0.016
68	*UVRAG*	11	75815210	76144232	Adrenal Gland	1.971	0.049	0.013
69	*PWP2*	21	44107399	44131181	Adrenal Gland	1.967	0.049	0.002

### Functional analysis

**Fig. 3:**
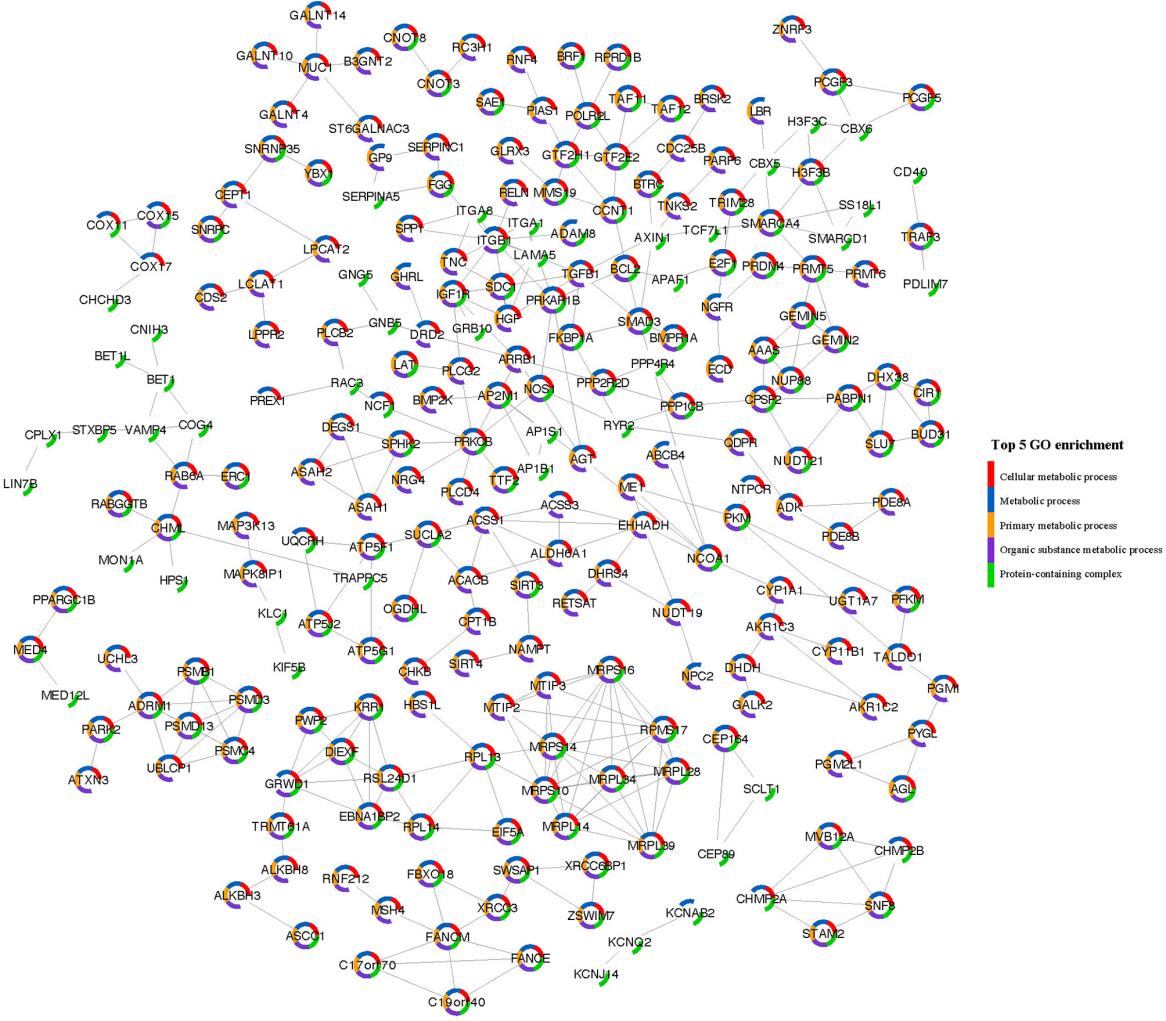
Protein‒protein interaction network of the TWAS-identified genes associated with SSBP. Each node represents one gene, and the edge represents the interaction relationships between two genes. The top 5 GO enrichment results are shown in 5 colours around the nodes

Cell-type enrichment analysis revealed that blood, muscle, eye, pancreas, spleen, bone marrow, large intestine, bladder, small intestine, lung, and tongue were the top 10 tissues in which the genes were enriched (Supplementary Fig. 2a). Monocytes, mesenchymal stem cells, epithelial cells, pancreatic ductal cells, myeloid cells, granulocytes, fibroblasts, enterocytes, macrophages, and erythroid progenitor cells were the top 10 general cell types (Supplementary Fig. 2b). The SPRING database showed that the genes significantly associated with the TWAS were mostly enriched in metabolic-related biological processes. There were 435 GO terms and 74 KEGG pathways associated with significant SSBP genes. The top 5 significant GO terms were cellular metabolic process (GO: 0044237, FDR = 1.75e-35), metabolic process (GO: 0008152, FDR = 4.85e-35), primary metabolic process (GO: 0044238, FDR = 5.21e-33), organic substance metabolic process (GO: 0071701, FDR = 5.21e-33) and protein-containing complex (GO: 0032991, FDR = 3.92e-31). The top 5 KEGG pathways were metabolic pathways (FDR = 6.35e-10), focal adhesion (FDR = 3.80e-04), ECM-receptor interaction (FDR = 3.80e-04), propanoate metabolism (FDR = 7.50e-04) and ribosome (FDR = 7.50e-04). The PPI network and the top 5 GO terms are shown in Fig. 3. The network contained 244 nodes and 353 edges after removing the genes that were isolated from the network.

### PRS and PTRS scoring

In total, 2,589 SNPs in the TWAS *P* < 0.05 subset, 621 SNPs in the TWAS *P* < 0.01 subset, 82 SNPs in the TWAS *P* < 0.001 subset and 10 SNPs in the *P* < 0.0001 subset were identified from the TWAS analysis results. After mapping the rsID with the “ped” document and the GWAS association results, 371, 84 and 10 SNPs were finally used to calculate the PRSs with TWAS *P* value of 0.05, 0.01 and 0.001, respectively. Additionally, 2,605 genes in the *P* < 0.05 subset, 585 genes in the *P* < 0.01 subset, 66 genes in the *P* < 0.001 subset, 7 genes in the *P* < 0.0001 subset and 69 DEGs were used to calculate the aggregate- and tissue-specific PTRS. The correlation matrix showed that the PRSs of 0.05, 0.01 and 0.001 had greater correlations with PTRS of 0.0001 than with the other PTRSs subsets. The correlation coefficients were 0.140, 0.142 and 0.266, respectively. The combinations of a PRS of 0.05, 0.01, and 0.001 with a PTRS of 0.0001 were further calculated to evaluate the prediction probabilities. Furthermore, the correlations between PRSs and tissue-specific PTRSs were also explored. We found that a PTRS of 0.05 in the heart atrial appendage was significantly associated with PRSs of 0.05, 0.01 and 0.001 (*P* < 0.05). Similarly, PTRS of 0.01 in the heart atrial appendage, PTRS of 69 DEGs in the adrenal gland, EBV-transformed lymphocytes in the pituitary gland, PTRS of 0.001 in the adrenal gland, artery coronary region, artery tibial region, whole blood and PTRS of 0.0001 in the adrenal gland were significantly associated with PRSs (Supplementary Figs. 3–7).

### Construction of SSBP risk evaluation models with PRSs and PTRSs

In the training dataset (*n* = 1176), the PRS quartiles were significantly positively associated with SSBP. The predictive abilities of the models increased after adjusting for covariates (age, sex, BMI, education, smoking status, diabetes status, hypertension status). The models with a PRS of 0.05 showed the best predictive ability (R^2^ = 0.329, AUC = 0.868, 95% CI: 0.845–0.890) compared with the PRS of 0.01 (R^2^ = 0.176, AUC = 0.765, 95% CI: 0.735–0.795) and PRS of 0.001 (R^2^ = 0.059, AUC = 0.646, 95% CI: 0.611–0.682). The results were also stable in the validation dataset (*n* = 491). The results of the validation dataset are shown in Supplementary Fig. 8. In addition, the 10-fold cross-validation suggested that the accuracy of the model with PRS of 0.05 was 82.16% and the AUC was 0.868 (95% CI: 0.861–0.875).

**Fig. 4:**
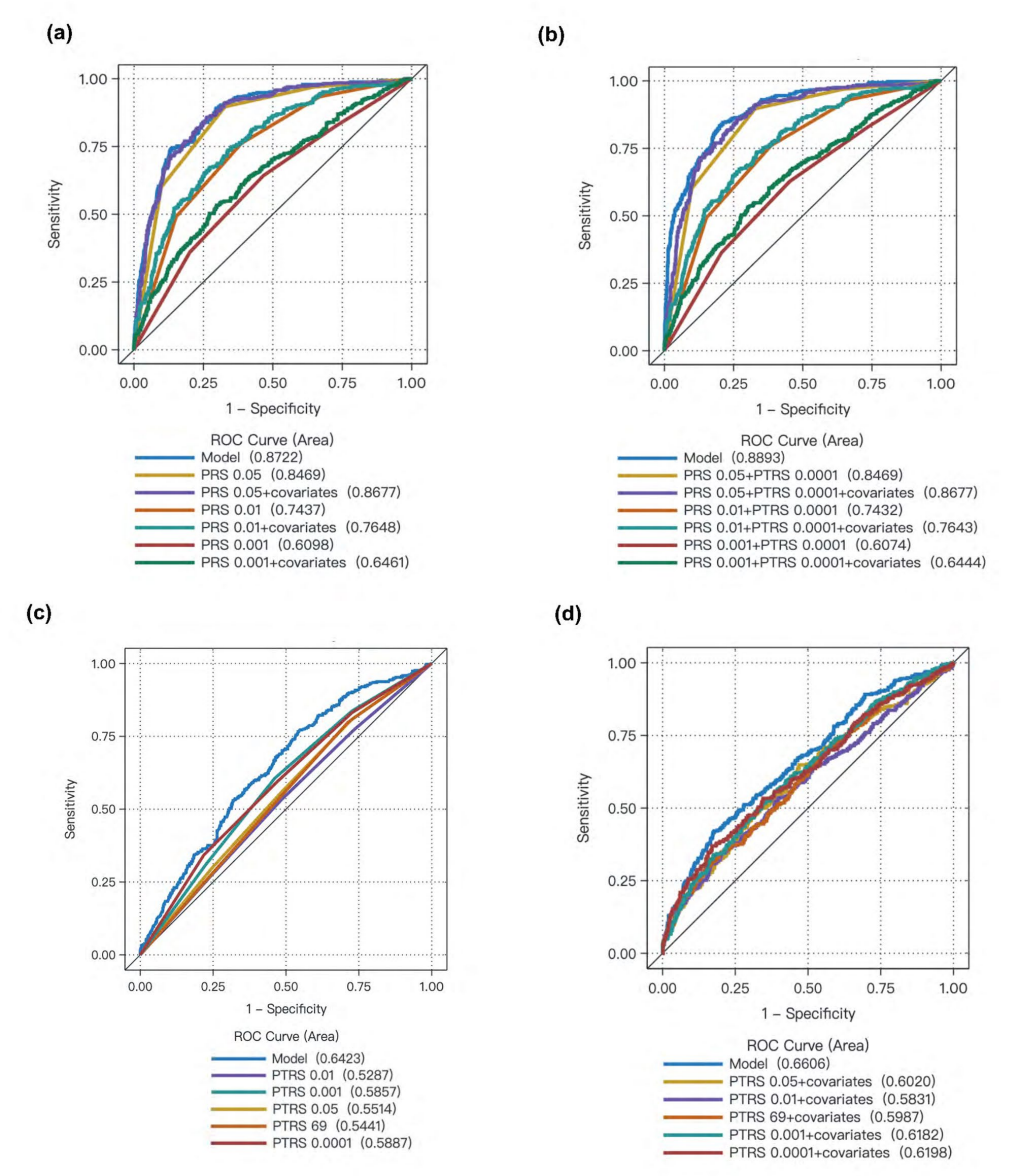
Receiver operating characteristic curves and areas under the curves of the SSBP risk evaluation models with PRSs (a), PRSs and PTRSs combinations (b), PTRSs with no covariates (c) and PTRSs with covariates (d) in the training dataset

Furthermore, the PTRS of 0.001, 0.0001 and 69 DEG quartiles showed significant positive associations with SSBP (Supplementary Table 3). PTRS of 0.0001 (R^2^ = 0.046, AUC = 0.620, 95% CI: 0.583–0.657) had a greater AUC than PTRS of 0.05 (R^2^ = 0.032, AUC = 0.602, 95% CI: 0.565-0.639), 0.01 (R^2^ = 0.027, AUC = 0.583, 95% CI: 0.546-0.621), 0.001 (R^2^ = 0.043, AUC = 0.618, 95% CI: 0.582-0.654) and PTRS of 69 DEGs (R^2^ = 0.034, AUC = 0.599, 95% CI: 0.562–0.635). However, the prediction accuracy of the PTRS was lower than that of the PRS. We also compared the performance of the PRS with that of the PRS and PTRS combinations. The results showed that the prediction accuracy of the PRS did not change greatly after combination with PTRS (Fig. 4).

Tissue-specific PTRS indicated that PTRS of 0.05 in the heart left ventricle (AUC = 0.597, 95% CI: 0.567–0.627), 0.01 in the pituitary (AUC = 0.595, 95% CI: 0.564–0.625) and 0.001 in the adrenal gland (AUC = 0.588, 95% CI: 0.558–0.617) had greater AUCs than other PTRS subsets. The ROC curves of the 12 tissue-specific PTRSs had greater AUCs than the single-tissue models (Supplementary Table 4).

## Discussion

SSBP is the genetic basis of the association between salt and hypertension. Previous studies used to identify genetic variants associated with SSBP, but the ability to interpret the relationships between genes and SSBP was limited [32]. TWAS could identify the gene-trait correlations based on the eQTL expression reference panels [33]. In this study, a TWAS analysis was firstly conducted to seek the susceptibility genes of SSBP from GWAS datasets. Then, the differences of genes between SS and SR were verified by mRNA microarray. In addition, the PRS, PTRS and their combinations were computed based on the susceptibility genes and their significant variants. Finally, the risk evaluation model of SSBP was established and the performance of the model was tested in the training and validation datasets. Our study provided important evidence that multiple genes were significantly associated with SSBP, and the genetic variants of susceptibility genes could be effectively used to evaluate the genetic risk of SSBP.

Among the susceptibility genes, several have been reported to be associated with SSBP. *CYP11B1*, also known as cytochrome P450 family 11 subfamily B member 1 (11β-hydroxylase), is involved in the conversion of progesterone to cortisol in the adrenal cortex. Montasser et al. found that SNPs in *CYP11B1* were associated with the glomerular filtration rate (GFR) in the GenSalt study [34]. However, compared to *CYP11B1*, the C-344T polymorphism in the *CYP11B2* gene is significantly associated with urinary sodium excretion and affects salt sensitivity in Japanese individuals [35]. Similarly, in the present study, arrestin beta 1 (*ARRB1)* also exhibited significant associations with SSBP in TWASs according to microarray analysis. As a member of the arrestin/beta-arrestin protein family, *ARRB1* participates in agonist-mediated desensitization of G protein-coupled receptors (GPCRs) [36]. *ARRB1* can promote angiotensin II type 1 receptor (*AT1R*)-dependent aldosterone production [37] and is a key regulator of Na^+^/H^+^ exchangers [38]. Additionally, Sun et al. revealed that the overexpression of *ARRB1* in the rostral ventrolateral medulla downregulated the expression of *AT1R* and lowered blood pressure [39]. However, Mathieu et al. reported that *ARRB2*, rather than *ARRB1*, might counterbalance the canonical signalling of GPCRs in salt-sensitive hypertension [40]. More population research will be needed to explore the therapeutic role of *ARRB1* in SSBP.

Notably, we also identified several novel genes associated with SSBP. *EIF5A, H3-3 A, INTS1*, and *SEPTIN7P14* were widely expressed in all 12 tissues. Eukaryotic translation initiation Factor 5 A (*EIF5A*) is important for the synthesis of peptide bonds between consecutive proline residues and can enable the binding of U6 small nuclear RNA and protein N-terminus [41]. GO enrichment indicated that *EIF5A* was involved in several cellular processes, such as translational elongation, the regulation of transcription by RNA polymerase II, and the tumour necrosis factor-mediated signalling pathway, which were associated with cancer [42], renal ischaemia [43] and neurodegenerative disorders [44]. *H3-3 A* is a replication-independent member of the histone H3 family that is linked to cell proliferation, muscle cell differentiation, cell growth and nucleosome assembly [45]. *INTS1* is a subunit of the integrator complex and plays roles in gene expression, RNA polymerase II transcription and 3-prime end processing of snRNA. Septin 7 pseudogene 14 (*SEPTIN7P14*) is a pseudogene that does not have protein-coding ability. Although associations of the four genes with SSBP have not been reported, our results could provide insights into their potential roles in the pathogenesis of SSBP.

In this study, the SSBP susceptibility genes were found to be enriched in metabolic process pathways. This finding is consistent with other studies. Shi et al. identified novel metabolites associated with SSBP and hypertension using untargeted metabolomics in the GenSalt study. They found that serine, 2-methylbutyrylcarnitine and isoleucine were directly associated with high salt sensitivity in a dietary intervention trial [46]. To explore the metabolites associated with SSBP using the acute oral load and diuresis shrinkage test, our team conducted an untargeted metabolomics analysis in the EpiSS study and discovered the best performance of L-glutamine in the diagnosis of SSBP [47]. Nitric oxide synthase, oxidative stress, inflammatory reactions, and gut microbes may be potential mechanisms by which metabolites play a part in the control of SSBP [48–50]. Recently, Muller et al. reported that Na^+^ regulated the energy metabolism of immune cells in salt sensitivity [51]. The understanding of immunometabolism in SSBP may extend the definition of SSBP from different viewpoints.

Considering the difficulties of clinical diagnosis, multiple auxiliary diagnostic models have been developed based on biomarkers of SSBP. The biomarkers varied from candidate genetic predispositions [10, 52] and gene expression [27] to noncoding RNAs [53–55] and metabolites [47]. Among them, SNPs have the advantages of high gene density, good genetic stability, easy detection and are suitable for large-scale analysis. PRSs are built based on SNPs, so the risk of disease can be predicted in early life, and preventive measures can be taken in a timely manner for high-risk individuals [9]. Liu et al. used 42 known SNPs to calculate the PRS and estimate the joint effects of the SNPs on SSBP. They found that participants with higher PRS performed a more than twofold increased risk of SSBP [10]. However, they did not evaluate the effects of PRS on the diagnosis of SSBP. In this study, we used PRS or PTRS to construct a risk evaluation model for SSBP to distinguish SS from SR. By genotyping 307 SNPs, this model can be used for early identification of SS individuals in clinic. Considering the reduction of the cost of SNP chip, this model will have broad prospect in clinical application. Additionally, the PRS that based on the susceptibility genes of SSBP could explain more genetic information than the PRS that calculated from GWAS analysis. Moreover, PRS showed better performance than the PTRS in this study, which is consistent with the results of other studies [21]. Although PTRS does not outperform PRS, it is still a promising approach that uses the gene expression of trait-associated genes from GWASs, which are closer to traits than SNPs. At the same time, we found that PTRS was correlated with PRS, especially in the heart atrial appendage, adrenal gland, EBV-transformed lymphocytes, pituitary, artery coronary, artery tibial and whole blood. This may be due to the involvement of these tissues in the pathogenesis of SSBP [56, 57]. We did not observe a significant increase in the prediction accuracy of the PRS and PTRS combination, which might be attributed to the following reasons: (1) The PTRS is calculated using the predicted gene expression rather than the detected expression, so it may affect the prediction effect; (2) The number of genes in the PTRS subset is less than the SNPs in PRS, so the role of PTRS is limited in the evaluation of SSBP; (3) In the combined analysis of PTRS and PRS, we use the PCA to calculate the weight coefficients of PTRS and PRS. Because PRS is greater than PTRS, the combination scores are mainly affected by PRS. Other combination methods need to be developed in the future to optimize the results. Furthermore, GTEx v8 reference panels of all ancestry samples were used to predict the SSBP-associated genes. The portability of the PTRS of SSBP across ancestries needs to be further explored with multiple ancestry group collaborations.

Our study has the strengths of investigating the genetic predisposition of SSBP based on the strictly established EpiSS cohort, comprehensively identifying the susceptibility genes of genes in multiple tissues and validating the genes in a microarray to improve the generalizability of the results. Additionally, the TWAS results were transformed into PRSs and PTRSs and were used to construct a risk evaluation model of SSBP to facilitate clinical application. However, this study still has several limitations. First, due to the difficulties of large-scale SSBP diagnosis and the diversity of diagnostic methods, compared to other chronic diseases, the sample size was relatively small. In the future, team cooperation is vital for expanding the sample size, improving the statistical power and validating the present results in different populations. Second, considering that the TWAS analysis is based on statistical prediction, the causal effects of susceptibility genes on SSBP are still uncertain. Further functional experiments are needed to verify the causality of genes related to SSBP. Third, the reference panel of the TWAS analysis was European-derived, so the results should be interpreted with caution. Fourth, the participants in this study were mainly middle-aged adults with a greater burden of chronic noncommunicable diseases, which limits the generalizability of the results. Finally, only the cis-genetic component of gene expression was considered in the current TWAS analysis. An algorithm that can be used to investigate trans-eQTL effects is needed in future research.

In conclusion, several known and novel susceptibility genes for SSBP were identified via multitissue TWAS analysis. The risk evaluation model based on genetic variations in susceptibility genes showed good diagnostic performance and could be used in clinics and communities to screen high-risk individuals for SSBP.

### Electronic supplementary material

Below is the link to the electronic supplementary material.

Supplementary Material 1

## Data Availability

The datasets generated during and/or analysed during the current study are available from the corresponding author upon reasonable request.
