# Supplementary material for: Susceptibility gene identification and risk evaluation model construction by transcriptome-wide association analysis for salt sensitivity of blood pressure
Source: BMC Genomics. 2024 Jun 18;25:612. doi: 10.1186/s12864-024-10409-9 (PMC11184770; doi:10.1186/s12864-024-10409-9)
Supplement: Supplementary file 1 — Supplementary Material 1 [file 12864_2024_10409_MOESM1_ESM.docx]

**Supplementary Material**

- **Supplementary Table 1.** Significant SNPs in GWAS analysis (P<1.00e-05).
- **Supplementary Table 2.** Jointly significant genes in conditional analysis.
- **Supplementary Table 3.** The associations of PTRS with different P thresholds with SSBP in the binary logistic regressions.
- **Supplementary Table 4.** ROC association statistics of 12 tissue specific PTRS with different P thresholds in the associations with SSBP.
- **Supplementary Figure 1.** The Manhattan plot of GWAS analysis.
- **Supplementary Figure 2.** Top 20 Cell-type (a) and (b) tissues enrichment results.
- **Supplementary Figure 3.** The heat map of the Spearman correlation matrix of PRS 0.05, 0.01, 0.001 and 12 tissue specific PTRS of P<0.05 scores.
- **Supplementary Figure 4.** The heat map of the Spearman correlation matrix of PRS 0.05, 0.01, 0.001 and 12 tissue specific PTRS of P<0.01 scores.
- **Supplementary Figure 5.** The heat map of the Spearman correlation matrix of PRS 0.05, 0.01, 0.001 and 12 tissue specific PTRS of 69 differently expressed genes.
- **Supplementary Figure 6.** The heat map of the Spearman correlation matrix of PRS 0.05, 0.01, 0.001 and 12 tissue specific PTRS of P<0.001 scores.
- **Supplementary Figure 7.** The heat map of the Spearman correlation matrix of PRS 0.05, 0.01, 0.001 and 12 tissue specific PTRS of P<0.0001 scores.
- **Supplementary Figure 8.** The ROCs and AUCs of SSBP risk evaluations models with PRS (a), PRS+PTRS (b), PTRS with no covariates (c) and PTRS with covariates (d) in validation dataset.

**Supplementary Table 1. Significant SNPs in GWAS analysis (*P*<1×10^-5^).**

| **CHR** | **SNP** | **BP** | **A1** | **A2** | **MAF** | **OR** | **SE** | **L95** | **U95** | **STAT** | ***P* value** |
| --- | --- | --- | --- | --- | --- | --- | --- | --- | --- | --- | --- |
| 1 | rs4244632 | 9670483 | T | A | 0.288 | 1.469 | 0.085 | 1.244 | 1.735 | 4.531 | 5.86×10^-6^ |
| 1 | rs4450053 | 9673779 | C | A | 0.288 | 1.471 | 0.085 | 1.245 | 1.737 | 4.543 | 5.53×10^-6^ |
| 1 | rs17460395 | 102180213 | A | G | 0.029 | 2.692 | 0.218 | 1.758 | 4.124 | 4.554 | 5.25×10^-6^ |
| 1 | rs1373821 | 102181823 | T | C | 0.029 | 2.738 | 0.218 | 1.788 | 4.193 | 4.632 | 3.63×10^-6^ |
| 1 | rs56025619 | 102190446 | G | A | 0.029 | 2.626 | 0.216 | 1.721 | 4.007 | 4.479 | 7.57×10^-6^ |
| 1 | rs17426819 | 102194625 | G | A | 0.029 | 2.661 | 0.214 | 1.748 | 4.051 | 4.565 | 4.98×10^-6^ |
| 1 | rs2044368 | 102196723 | G | A | 0.032 | 2.514 | 0.207 | 1.678 | 3.769 | 4.465 | 7.99×10^-6^ |
| 1 | rs72988810 | 102197021 | A | T | 0.029 | 2.729 | 0.215 | 1.789 | 4.162 | 4.660 | 3.16×10^-6^ |
| 1 | rs10493962 | 102222006 | G | C | 0.030 | 2.605 | 0.214 | 1.714 | 3.958 | 4.485 | 7.31×10^-6^ |
| 1 | rs959164 | 102228702 | G | C | 0.030 | 2.670 | 0.215 | 1.753 | 4.065 | 4.578 | 4.69×10^-6^ |
| 3 | rs400048 | 1587860 | T | C | 0.171 | 0.612 | 0.111 | 0.493 | 0.761 | -4.432 | 9.33×10^-6^ |
| 3 | rs80017671 | 40330427 | C | T | 0.088 | 0.487 | 0.161 | 0.356 | 0.668 | -4.463 | 8.09×10^-6^ |
| 5 | rs10472795 | 31893865 | A | G | 0.313 | 0.672 | 0.087 | 0.567 | 0.796 | -4.591 | 4.41×10^-6^ |
| 6 | rs12192595 | 83504681 | C | T | 0.048 | 2.162 | 0.168 | 1.554 | 3.008 | 4.579 | 4.67×10^-6^ |
| 8 | rs143884031 | 11535853 | C | T | 0.017 | 3.572 | 0.280 | 2.062 | 6.188 | 4.542 | 5.57×10^-6^ |
| 9 | rs1327046 | 22241938 | T | C | 0.053 | 0.354 | 0.227 | 0.227 | 0.552 | -4.574 | 4.78×10^-6^ |
| 9 | rs1751450 | 22242502 | A | G | 0.055 | 0.364 | 0.219 | 0.237 | 0.600 | -4.605 | 4.13×10^-6^ |
| 10 | rs11000477 | 74730322 | C | T | 0.075 | 1.850 | 0.139 | 1.409 | 2.427 | 4.433 | 9.28×10^-6^ |
| 10 | rs7904721 | 74731598 | T | C | 0.075 | 1.868 | 0.138 | 1.424 | 2.451 | 4.516 | 6.31×10^-6^ |
| 10 | rs4745708 | 74732448 | C | T | 0.075 | 1.850 | 0.139 | 1.409 | 2.427 | 4.434 | 9.26×10^-6^ |
| 14 | rs2333109 | 28259506 | T | C | 0.144 | 0.553 | 0.125 | 0.433 | 0.706 | -4.746 | 2.07×10^-6^ |
| 14 | rs147397904 | 28262085 | C | T | 0.140 | 0.548 | 0.126 | 0.428 | 0.701 | -4.775 | 1.80×10^-6^ |
| 14 | rs117384687 | 28263381 | A | G | 0.139 | 0.550 | 0.126 | 0.430 | 0.704 | -4.741 | 2.13×10^-6^ |
| 14 | rs10137169 | 28297700 | G | A | 0.139 | 0.570 | 0.125 | 0.446 | 0.728 | -4.498 | 6.85×10^-6^ |
| 15 | rs4776576 | 72098672 | A | G | 0.109 | 1.686 | 0.118 | 1.339 | 2.122 | 4.439 | 9.03×10^-6^ |
| 18 | rs8090057 | 11458810 | T | C | 0.148 | 1.610 | 0.106 | 1.308 | 1.981 | 4.498 | 6.85×10^-6^ |
| 18 | rs1455243 | 11459465 | G | A | 0.148 | 1.610 | 0.106 | 1.308 | 1.981 | 4.498 | 6.85×10^-6^ |
| 18 | rs66472215 | 11460110 | T | C | 0.148 | 1.613 | 0.106 | 1.311 | 1.985 | 4.518 | 6.25×10^-6^ |
| 18 | rs12968933 | 11462309 | A | G | 0.148 | 1.609 | 0.106 | 1.308 | 1.980 | 4.495 | 6.95×10^-6^ |
| 18 | rs35229964 | 11464533 | T | C | 0.148 | 1.613 | 0.106 | 1.311 | 1.985 | 4.520 | 6.20×10^-6^ |
| 18 | rs2198663 | 11468379 | C | G | 0.148 | 1.609 | 0.106 | 1.308 | 1.979 | 4.497 | 6.89×10^-6^ |
| 18 | rs34927495 | 11468636 | A | G | 0.148 | 1.635 | 0.105 | 1.331 | 2.009 | 4.677 | 2.92×10^-6^ |
| 18 | rs7228356 | 11471510 | T | G | 0.142 | 1.676 | 0.107 | 1.359 | 2.068 | 4.822 | 1.43×10^-6^ |
| 18 | rs8089262 | 11472697 | T | C | 0.149 | 1.666 | 0.104 | 1.358 | 2.045 | 4.892 | 1.00×10^-6^ |
| 18 | rs6505655 | 11475735 | G | A | 0.149 | 1.633 | 0.105 | 1.328 | 2.007 | 4.652 | 3.30×10^-6^ |
| 21 | rs138139129 | 42892518 | C | T | 0.039 | 0.286 | 0.282 | 0.165 | 0.498 | -4.430 | 9.43×10^-6^ |

Note: CHR, chromosome; SNP, single nucleotide polymorphisms; A1, minor allele; A2, major allele; MAF, minor allele frequency; OR, odds ratio; SE, standard error; L95, lower limmit of 95% confidence intervals; U95, upper limmit of 95% confidence intervals; STAT, statistic values.

**Supplementary Table 2. Jointly significant genes in conditional analysis.**

| **GTEx Weight Set** | **Joint significant genes** | **Location** | **No of Marginally significant genes** | **JOINT.Z** | **JOINT.P** |
| --- | --- | --- | --- | --- | --- |
| Pituitary | TMEM201 | Chromosome 1: 9,588,911-9,614,877 | 2 | -3.8 | 1.40e-04 |
| Whole Blood | UBXN11 | Chromosome 1: 26,281,328-26,318,363 | 5 | -4.4 | 1.20e-05 |
| Adipose Subcutaneous | GNAQP1 | Chromosome 2: 131,423,801-131,424,867 | 3 | 3.7 | 2.20e-04 |
| Artery Aorta | ZNF300 | Chromosome 5: 150,894,392-150,904,983 | 4 | 3.6 | 3.30e-04 |
| Adipose Subcutaneous | TNFRSF11B | Chromosome 8: 118,923,557-118,951,885 | - | 3.5 | 4.00e-04 |
| Adrenal Gland | GTF2E2 | Chromosome 8: 30,578,318-30,658,236 | 2 | 4 | 7.40e-05 |
| Artery Coronary | ZNF510 | Chromosome 9: 96,754,553-96,778,129 | - | -2.4 | 1.70e-02 |
| Artery Coronary | ENSG00000273038 | Retired | 4 | 3.8 | 1.40e-04 |
| Heart Atrial Appendage | Novel gene | Chromosome 10: 73,098,044-73,101,297 | 13 | -4 | 5.60e-05 |
| Whole Blood | R3HCC1L | Chromosome 10: 98,134,624-98,244,897 | - | 3.7 | 2.00e-04 |
| Adipose Visceral Omentum | PKP3 | Chromosome 11: 392,614-404,908 | 13 | -3.5 | 4.50e-04 |
| Kidney Cortex | SRRM4 | Chromosome 12: 118,981,541-119,163,051 | 0 | -4.1 | 5.00e-05 |
| Adipose Visceral Omentum | IGHV4-59 | Chromosome 14: 106,627,249-106,627,825 | 9 | 3.7 | 0.20e-03 |
| Adrenal Gland | novel gene | Chromosome 14: 103,694,560-103,695,170 | 18 | -3.9 | 1.10e-03 |
| Adipose Visceral Omentum | GRAMD2A | Chromosome 15: 72,159,806-72,197,787 | 5 | -4.2 | 2.60e-05 |
| Heart Left Ventricle | ARHGAP17 | Chromosome 16: 24,919,389-25,015,666 | 6 | -3.9 | 1.00e-04 |
| Pituitary | KRT10-AS1 | Chromosome 17: 40,819,101-40,836,270 | 1 | -3.5 | 4.60e-04 |
| Pituitary | SLC14A1 | Chromosome 18: 45,687,025-45,752,520 | - | -3.4 | 5.90e-04 |
| Adipose Subcutaneous | LINC01260 | Chromosome 20: 44,656,451-44,663,498 | 2 | 3.7 | 0.20e-03 |
| Whole Blood | ZNF341-AS1 | Chromosome 20: 33,787,373-33,811,109 | 12 | 3.8 | 1.30e-04 |

**Supplementary Table 3. The associations of PTRS with different P thresholds with SSBP in the binary logistic regressions.**

| **PTRS** | **Category** | **SS**  **n (%)** | **OR (95% CI)** | **AOR (95% CI)** |
| --- | --- | --- | --- | --- |
| PTRS 0.05 | Q1 | 141 (28.72) | Ref | Ref |
|  | Q2 | 118 (24.03) | 0.772 (0.574-1.039) | 0.790 (0.585-1.067) |
|  | Q3 | 129 (26.27) | 0.889 (0.664-1.191) | 0.816 (0.605-1.102) |
|  | Q4 | 103 (20.98) | 0.660 (0.487-0.895) | 0.670 (0.493-0.912) |
| PTRS 0.01 | Q1 | 137 (27.9) | Ref | Ref |
|  | Q2 | 117 (23.83) | 0.802 (0.595-1.081) | 0.753 (0.555-1.023) |
|  | Q3 | 132 (26.88) | 0.960 (0.716-1.286) | 0.957 (0.711-1.287) |
|  | Q4 | 105 (21.38) | 0.685 (0.506-0.928) | 0.684 (0.503-0.929) |
| PTRS 0.001 | Q1 | 88 (17.92) | Ref | Ref |
|  | Q2 | 103 (20.98) | 1.224 (0.884-1.696) | 1.238 (0.890-1.723) |
|  | Q3 | 150 (30.55) | **2.083 (1.527-2.843)** | **1.983 (1.443-2.723)** |
|  | Q4 | 150 (30.55) | **2.106 (1.543-2.873)** | **2.117 (1.547-2.898)** |
| PTRS 69 | Q1 | 101 (20.57) | Ref | Ref |
|  | Q2 | 121 (24.64) | 1.343 (0.984-1.833) | 1.318 (0.962-1.805) |
|  | Q3 | 129 (26.27) | **1.459 (1.071-1.989)** | **1.388 (1.013-1.901)** |
|  | Q4 | 140 (28.51) | **1.639 (1.207-2.225)** | **1.642 (1.206-2.237)** |
| PTRS 0.0001 | Q1 | 87 (17.72) | Ref | Ref |
|  | Q2 | 113 (23.01) | 1.445 (1.047-1.995) | 1.368 (0.985-1.901) |
|  | Q3 | 129 (26.27) | **1.651 (1.202-2.268)** | **1.636 (1.186-2.257)** |
|  | Q4 | 162 (32.99) | **2.482 (1.819-3.386)** | **2.531 (1.849-3.464)** |

Note: PTRS Q1 was set as reference. OR, odds ratio; AOR, adjusted odds ratio that including the covariables (age, sex, BMI, smoking, hypertension and diabetes) in the models. Bold represented statistical significant associations of PTRS categories with SSBP (P <0.05).

**Supplementary Table 4. ROC association statistics of 12 tissue specific PTRS with different P thresholds in the associations with SSBP.**

| **PTRS** | **Number of genes** | **ROC model** | **AUC** | **SE** | **95% Wald CI** | | **Somers' D** | **Gamma** | **Tau-a** |
| --- | --- | --- | --- | --- | --- | --- | --- | --- | --- |
|  |  |  |  |  | **Lower** | **Upper** |  |  |  |
| PTRS 0.05 | 348 | Factor 1 | 0.562 | 0.015 | 0.533 | 0.592 | 0.125 | 0.125 | 0.052 |
|  | 282 | Factor 2 | 0.554 | 0.016 | 0.524 | 0.584 | 0.108 | 0.108 | 0.045 |
|  | 173 | Factor 3 | 0.535 | 0.016 | 0.505 | 0.565 | 0.070 | 0.070 | 0.029 |
|  | 274 | Factor 4 | 0.519 | 0.016 | 0.489 | 0.550 | 0.038 | 0.038 | 0.016 |
|  | 131 | Factor 5 | 0.542 | 0.015 | 0.512 | 0.572 | 0.083 | 0.083 | 0.034 |
|  | 331 | Factor 6 | 0.523 | 0.015 | 0.493 | 0.553 | 0.046 | 0.046 | 0.019 |
|  | 121 | Factor 7 | 0.511 | 0.016 | 0.481 | 0.542 | 0.022 | 0.022 | 0.009 |
|  | 222 | Factor 8 | 0.506 | 0.016 | 0.476 | 0.537 | 0.013 | 0.013 | 0.005 |
|  | 203 | Factor 9 | 0.597 | 0.016 | 0.567 | 0.627 | 0.194 | 0.194 | 0.080 |
|  | 48 | Factor 10 | 0.543 | 0.016 | 0.513 | 0.574 | 0.086 | 0.086 | 0.036 |
|  | 189 | Factor 11 | 0.506 | 0.015 | 0.476 | 0.536 | 0.012 | 0.012 | 0.005 |
|  | 283 | Factor 12 | 0.503 | 0.016 | 0.472 | 0.534 | 0.006 | 0.006 | 0.003 |
|  | 2605 | All | 0.660 | 0.015 | 0.631 | 0.689 | 0.319 | 0.319 | 0.132 |
|  | 2605 | All + covariables | 0.677 | 0.015 | 0.649 | 0.706 | 0.355 | 0.355 | 0.147 |
| PTRS 0.01 | 75 | Factor 1 | 0.584 | 0.015 | 0.554 | 0.613 | 0.167 | 0.167 | 0.069 |
|  | 59 | Factor 2 | 0.531 | 0.015 | 0.501 | 0.561 | 0.062 | 0.062 | 0.026 |
|  | 41 | Factor 3 | 0.516 | 0.016 | 0.485 | 0.546 | 0.031 | 0.031 | 0.013 |
|  | 61 | Factor 4 | 0.526 | 0.016 | 0.495 | 0.556 | 0.051 | 0.051 | 0.021 |
|  | 29 | Factor 5 | 0.568 | 0.015 | 0.538 | 0.598 | 0.136 | 0.136 | 0.056 |
|  | 70 | Factor 6 | 0.521 | 0.016 | 0.490 | 0.551 | 0.042 | 0.042 | 0.017 |
|  | 23 | Factor 7 | 0.516 | 0.016 | 0.486 | 0.547 | 0.033 | 0.033 | 0.014 |
|  | 49 | Factor 8 | 0.535 | 0.015 | 0.504 | 0.565 | 0.069 | 0.069 | 0.029 |
|  | 46 | Factor 9 | 0.529 | 0.015 | 0.499 | 0.559 | 0.059 | 0.059 | 0.024 |
|  | 16 | Factor 10 | 0.505 | 0.016 | 0.474 | 0.536 | 0.010 | 0.010 | 0.004 |
|  | 40 | Factor 11 | 0.595 | 0.015 | 0.564 | 0.625 | 0.189 | 0.189 | 0.078 |
|  | 76 | Factor 12 | 0.587 | 0.015 | 0.556 | 0.617 | 0.173 | 0.173 | 0.072 |
|  | 585 | All | 0.672 | 0.014 | 0.644 | 0.700 | 0.344 | 0.344 | 0.142 |
|  | 585 | All + covariables | 0.681 | 0.014 | 0.653 | 0.709 | 0.362 | 0.362 | 0.149 |
| PTRS 0.001 | 10 | Factor 1 | 0.586 | 0.015 | 0.556 | 0.616 | 0.172 | 0.172 | 0.071 |
|  | 6 | Factor 2 | 0.580 | 0.015 | 0.550 | 0.610 | 0.159 | 0.159 | 0.066 |
|  | 5 | Factor 3 | 0.588 | 0.015 | 0.558 | 0.617 | 0.175 | 0.175 | 0.072 |
|  | 9 | Factor 4 | 0.576 | 0.015 | 0.546 | 0.606 | 0.152 | 0.152 | 0.063 |
|  | 2 | Factor 5 | 0.564 | 0.015 | 0.535 | 0.594 | 0.128 | 0.133 | 0.053 |
|  | 7 | Factor 6 | 0.576 | 0.015 | 0.547 | 0.606 | 0.152 | 0.152 | 0.063 |
|  | 2 | Factor 7 | 0.585 | 0.015 | 0.555 | 0.614 | 0.169 | 0.179 | 0.070 |
|  | 6 | Factor 8 | 0.582 | 0.015 | 0.552 | 0.612 | 0.164 | 0.164 | 0.068 |
|  | 5 | Factor 9 | 0.566 | 0.015 | 0.535 | 0.596 | 0.131 | 0.131 | 0.054 |
|  | 1 | Factor 10 | 0.531 | 0.016 | 0.500 | 0.561 | 0.061 | 0.061 | 0.025 |
|  | 4 | Factor 11 | 0.581 | 0.015 | 0.551 | 0.611 | 0.162 | 0.162 | 0.067 |
|  | 9 | Factor 12 | 0.577 | 0.016 | 0.547 | 0.608 | 0.154 | 0.154 | 0.064 |
|  | 66 | All | 0.676 | 0.014 | 0.648 | 0.704 | 0.352 | 0.352 | 0.145 |
|  | 66 | All + covariables | 0.690 | 0.014 | 0.662 | 0.717 | 0.379 | 0.379 | 0.157 |
| PTRS 69 | 15 | Factor 1 | 0.522 | 0.015 | 0.492 | 0.552 | 0.043 | 0.043 | 0.018 |
|  | 7 | Factor 2 | 0.541 | 0.016 | 0.510 | 0.572 | 0.082 | 0.082 | 0.034 |
|  | 11 | Factor 3 | 0.554 | 0.015 | 0.524 | 0.584 | 0.109 | 0.109 | 0.045 |
|  | 8 | Factor 4 | 0.553 | 0.016 | 0.522 | 0.584 | 0.106 | 0.106 | 0.044 |
|  | 1 | Factor 5 | 0.512 | 0.013 | 0.487 | 0.537 | 0.024 | 0.051 | 0.010 |
|  | 5 | Factor 6 | 0.548 | 0.016 | 0.518 | 0.579 | 0.097 | 0.097 | 0.040 |
|  | 3 | Factor 7 | 0.569 | 0.016 | 0.539 | 0.599 | 0.138 | 0.138 | 0.057 |
|  | 3 | Factor 8 | 0.539 | 0.016 | 0.508 | 0.570 | 0.078 | 0.078 | 0.032 |
|  | 4 | Factor 9 | 0.554 | 0.016 | 0.524 | 0.585 | 0.109 | 0.109 | 0.045 |
|  | 1 | Factor 10 | 0.508 | 0.016 | 0.477 | 0.539 | 0.016 | 0.016 | 0.007 |
|  | 4 | Factor 11 | 0.538 | 0.016 | 0.508 | 0.569 | 0.077 | 0.077 | 0.032 |
|  | 7 | Factor 12 | 0.555 | 0.016 | 0.524 | 0.586 | 0.109 | 0.109 | 0.045 |
|  | 69 | All | 0.631 | 0.015 | 0.601 | 0.661 | 0.262 | 0.262 | 0.108 |
|  | 69 | All + covariables | 0.664 | 0.015 | 0.635 | 0.694 | 0.328 | 0.328 | 0.136 |
| PTRS 0.0001 | 1 | Factor 2 | 0.539 | 0.016 | 0.508 | 0.570 | 0.078 | 0.087 | 0.032 |
|  | 2 | Factor 3 | 0.562 | 0.015 | 0.532 | 0.592 | 0.123 | 0.124 | 0.051 |
|  | 2 | Factor 8 | 0.550 | 0.016 | 0.519 | 0.581 | 0.100 | 0.100 | 0.041 |
|  | 1 | Factor 10 | 0.531 | 0.016 | 0.500 | 0.561 | 0.061 | 0.061 | 0.025 |
|  | 1 | Factor 12 | 0.501 | 0.011 | 0.479 | 0.522 | 0.001 | 0.004 | 0.001 |
|  | 7 | All | 0.596 | 0.015 | 0.566 | 0.626 | 0.191 | 0.191 | 0.079 |
|  | 7 | All + covariables | 0.628 | 0.016 | 0.598 | 0.659 | 0.257 | 0.257 | 0.106 |

Note: Factor 1, Adipose subcutaneous; Factor 2, Adipose visceral omentum; Factor 3, Adrenal gland; Factor 4, Artery aorta; Factor 5, Artery coronary; Factor 6, Artery tibial; Factor 7, EBV-transformed lymphocytes; Factor 8, Heart atrial appendage; Factor 9, Heart left ventricle; Factor 10, Kidney cortex; Factor 11, Pituitary; Factor 12, Whole blood; All model, put all the tissue specific PTRS in the model; All + covariables, All model adjusted for covariables. ROC, Receiver operating characteristic curve; AUC, Area under the curve; SE, standard error; CI, confidence intervals; Somers' D, Gamma and Tau-a were statistics that reflected the predicted probabilities of the associations. The PTRS scores were treated as continuous variables in the models.


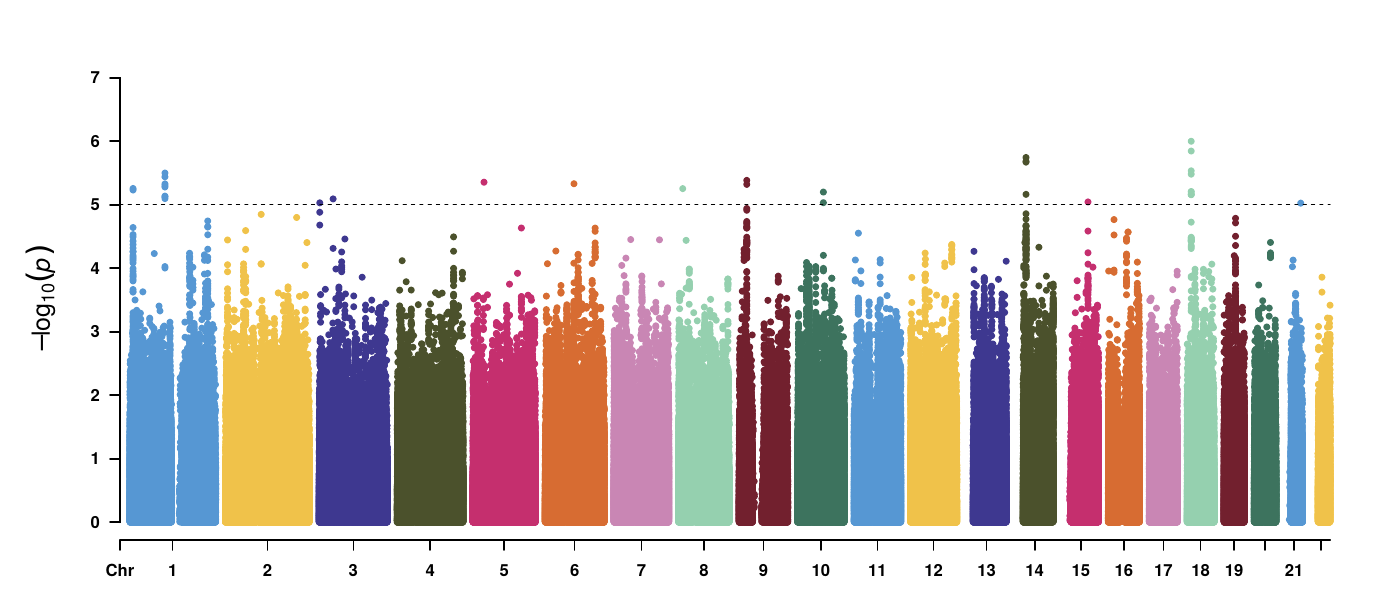


**Supplementary Figure 1. The Manhattan plot of GWAS analysis.**


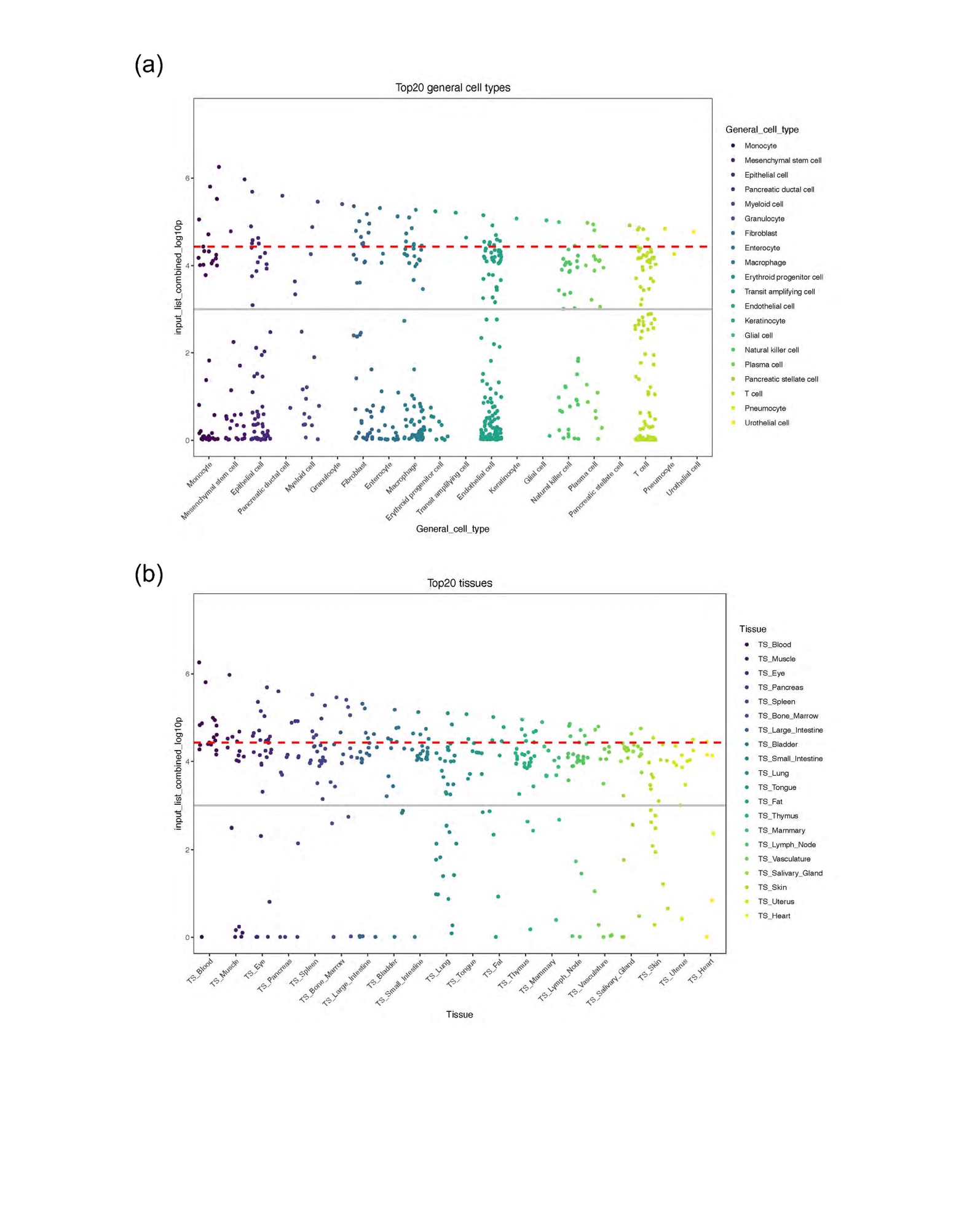


**Supplementary Figure 2. Top 20 Cell-type (a) and (b) tissues enrichment results.**

**
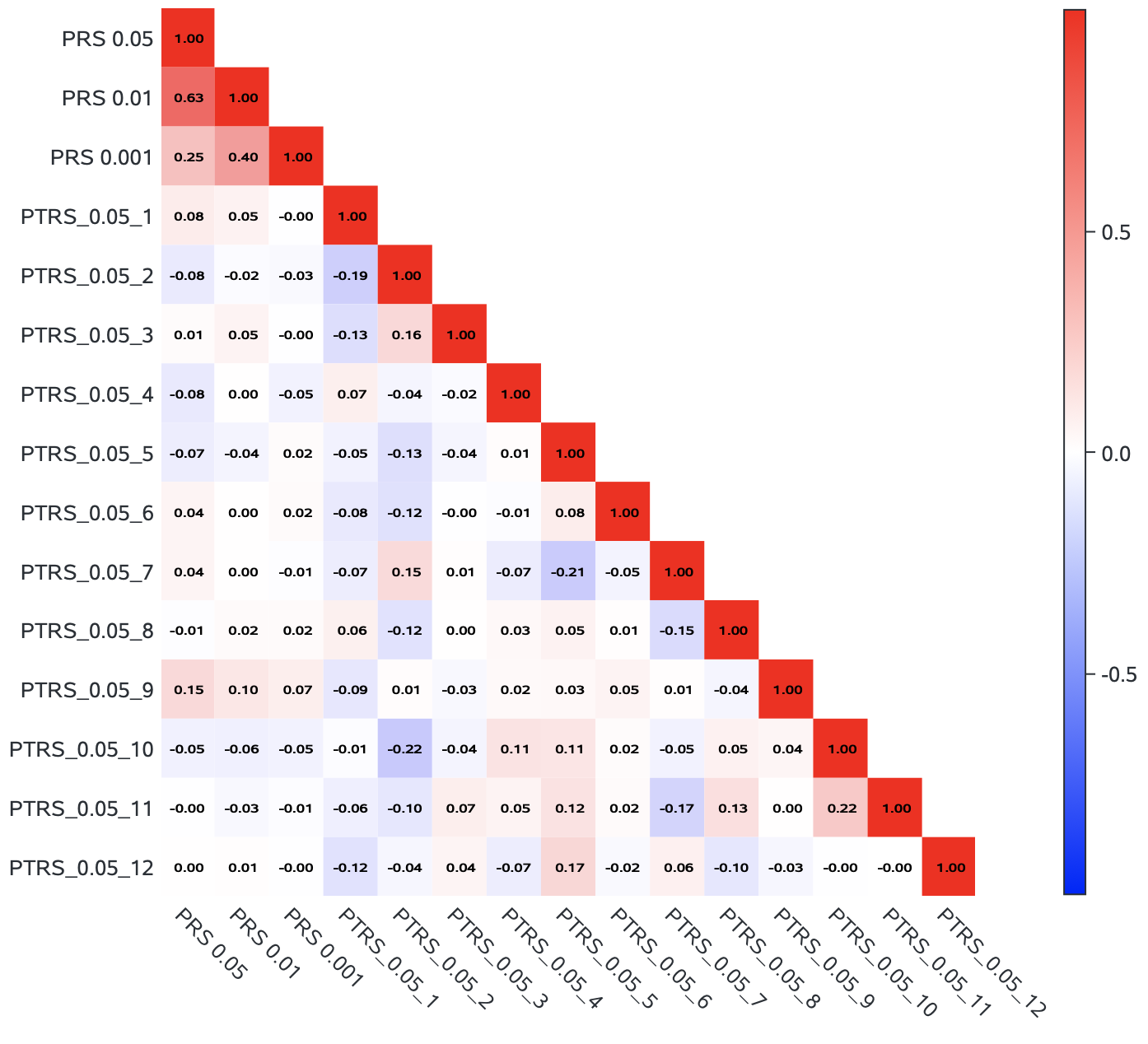
**

**Supplementary Figure 3. The heat map of the Spearman correlation matrix of PRS 0.05, 0.01, 0.001 and 12 tissue specific PTRS of P<0.05 scores.** 1-12 indicated Adipose subcutaneous, Adipose visceral omentum, Adrenal gland, Artery aorta, Artery coronary, Artery tibial, EBV-transformed lymphocytes, Heart atrial appendage, Heart left ventricle, Kidney cortex, Pituitary, Whole blood, respectively.

**
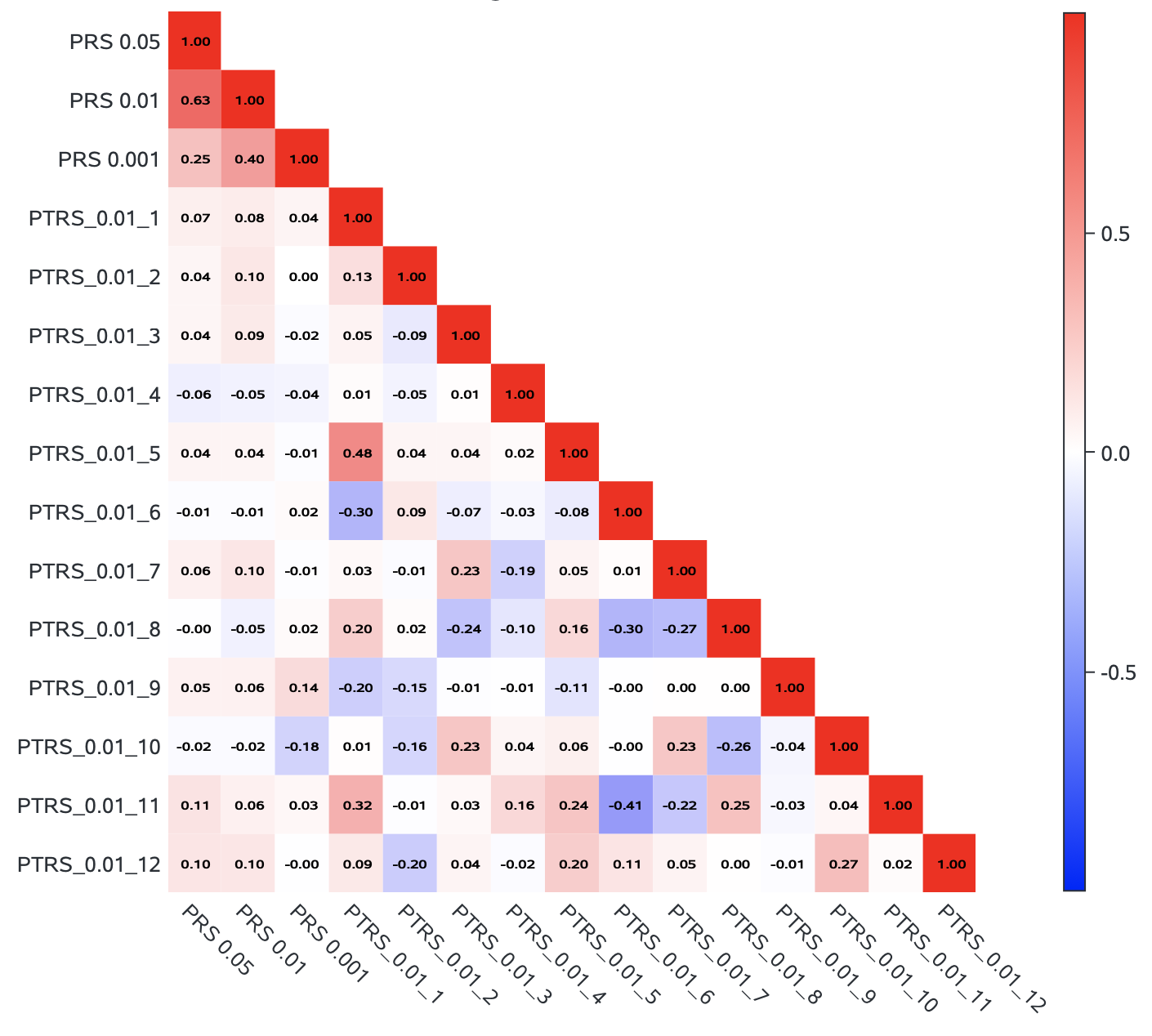
**

**Supplementary Figure 4. The heat map of the Spearman correlation matrix of PRS 0.05, 0.01, 0.001 and 12 tissue specific PTRS of P<0.01 scores.** 1-12 indicated Adipose subcutaneous, Adipose visceral omentum, Adrenal gland, Artery aorta, Artery coronary, Artery tibial, EBV-transformed lymphocytes, Heart atrial appendage, Heart left ventricle, Kidney cortex, Pituitary, Whole blood, respectively.

**
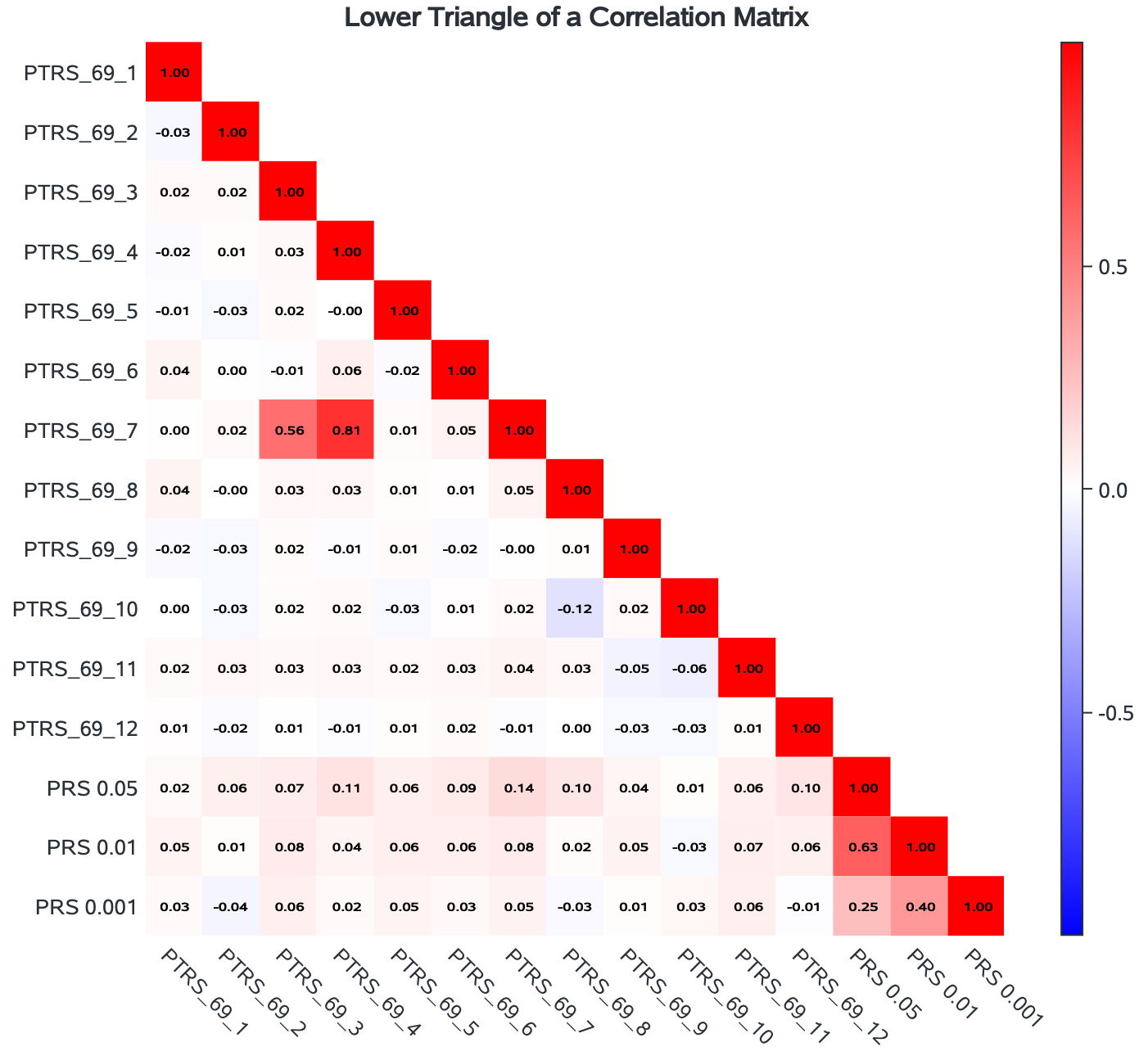
**

**Supplementary Figure 5. The heat map of the Spearman correlation matrix of PRS 0.05, 0.01, 0.001 and 12 tissue specific PTRS of 69 differently expressed genes (DEGs).** 1-12 indicated Adipose subcutaneous, Adipose visceral omentum, Adrenal gland, Artery aorta, Artery coronary, Artery tibial, EBV-transformed lymphocytes, Heart atrial appendage, Heart left ventricle, Kidney cortex, Pituitary, Whole blood, respectively.

**
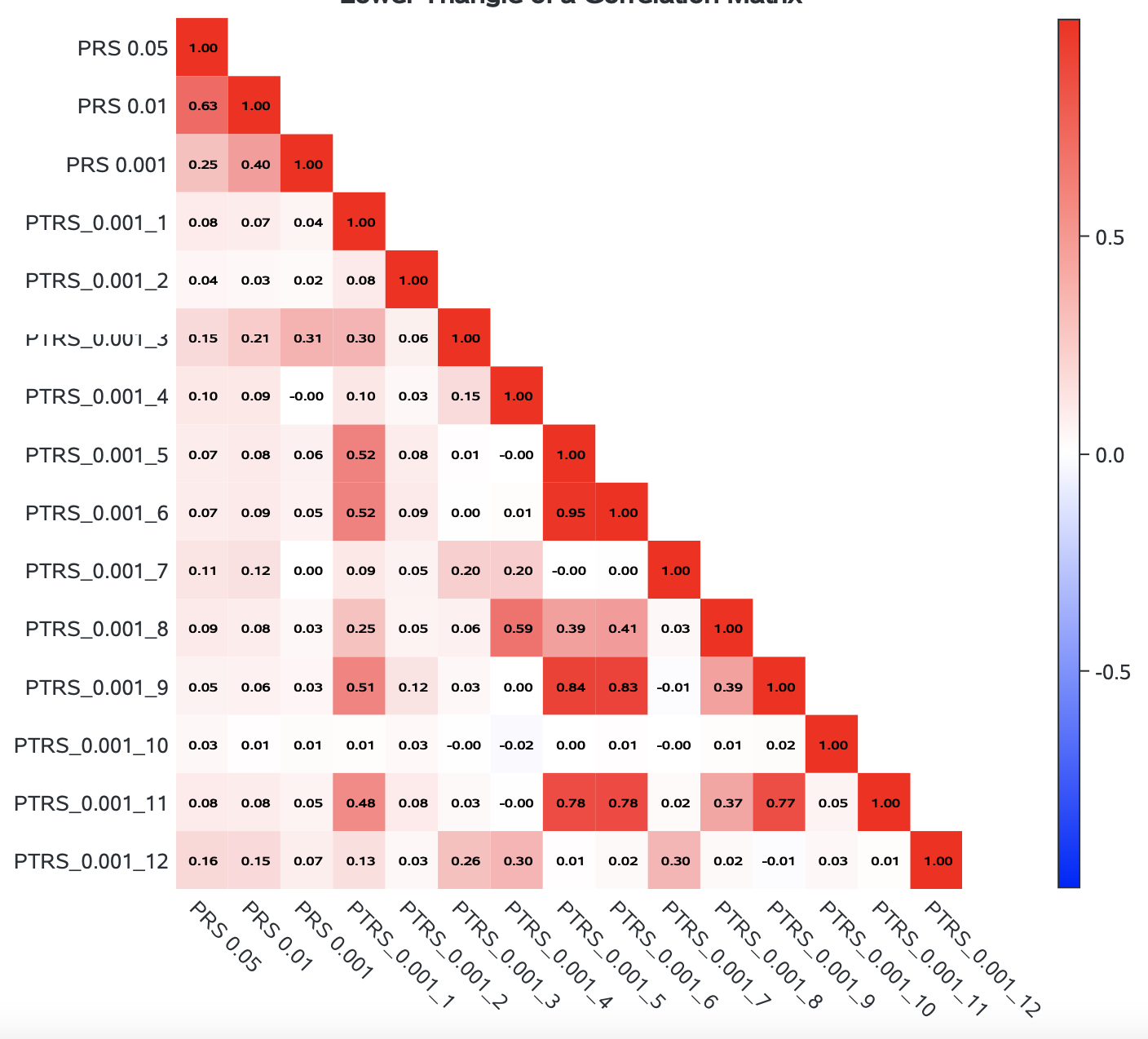
**

**Supplementary Figure 6. The heat map of the Spearman correlation matrix of PRS 0.05, 0.01, 0.001 and 12 tissue specific PTRS of P<0.001 scores.** 1-12 indicated Adipose subcutaneous, Adipose visceral omentum, Adrenal gland, Artery aorta, Artery coronary, Artery tibial, EBV-transformed lymphocytes, Heart atrial appendage, Heart left ventricle, Kidney cortex, Pituitary, Whole blood, respectively.

**
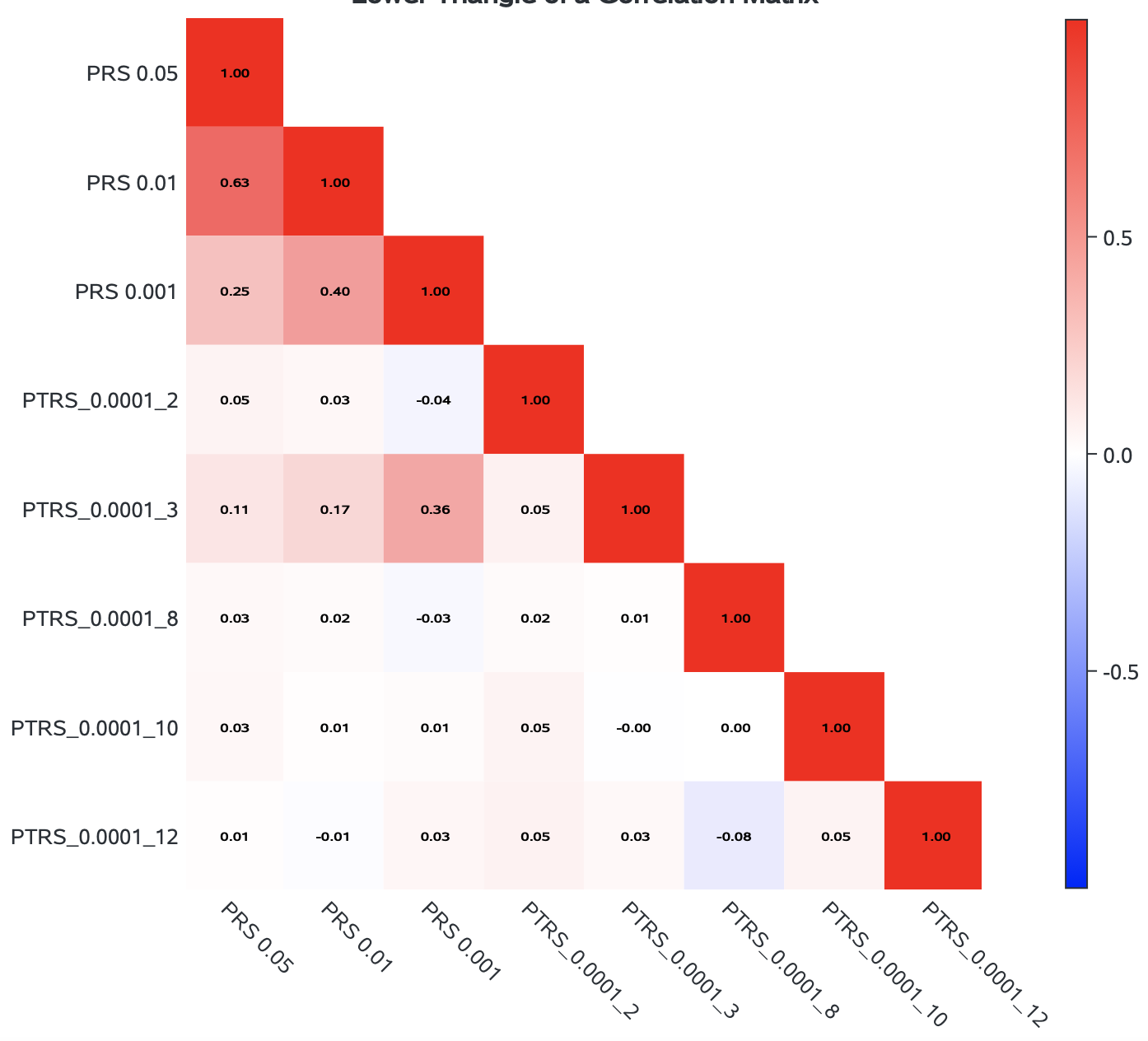
**

**Supplementary Figure 7. The heat map of the Spearman correlation matrix of PRS 0.05, 0.01, 0.001 and 12 tissue specific PTRS of P<0.0001 scores.** 1-12 indicated Adipose subcutaneous, Adipose visceral omentum, Adrenal gland, Artery aorta, Artery coronary, Artery tibial, EBV-transformed lymphocytes, Heart atrial appendage, Heart left ventricle, Kidney cortex, Pituitary, Whole blood, respectively.

**
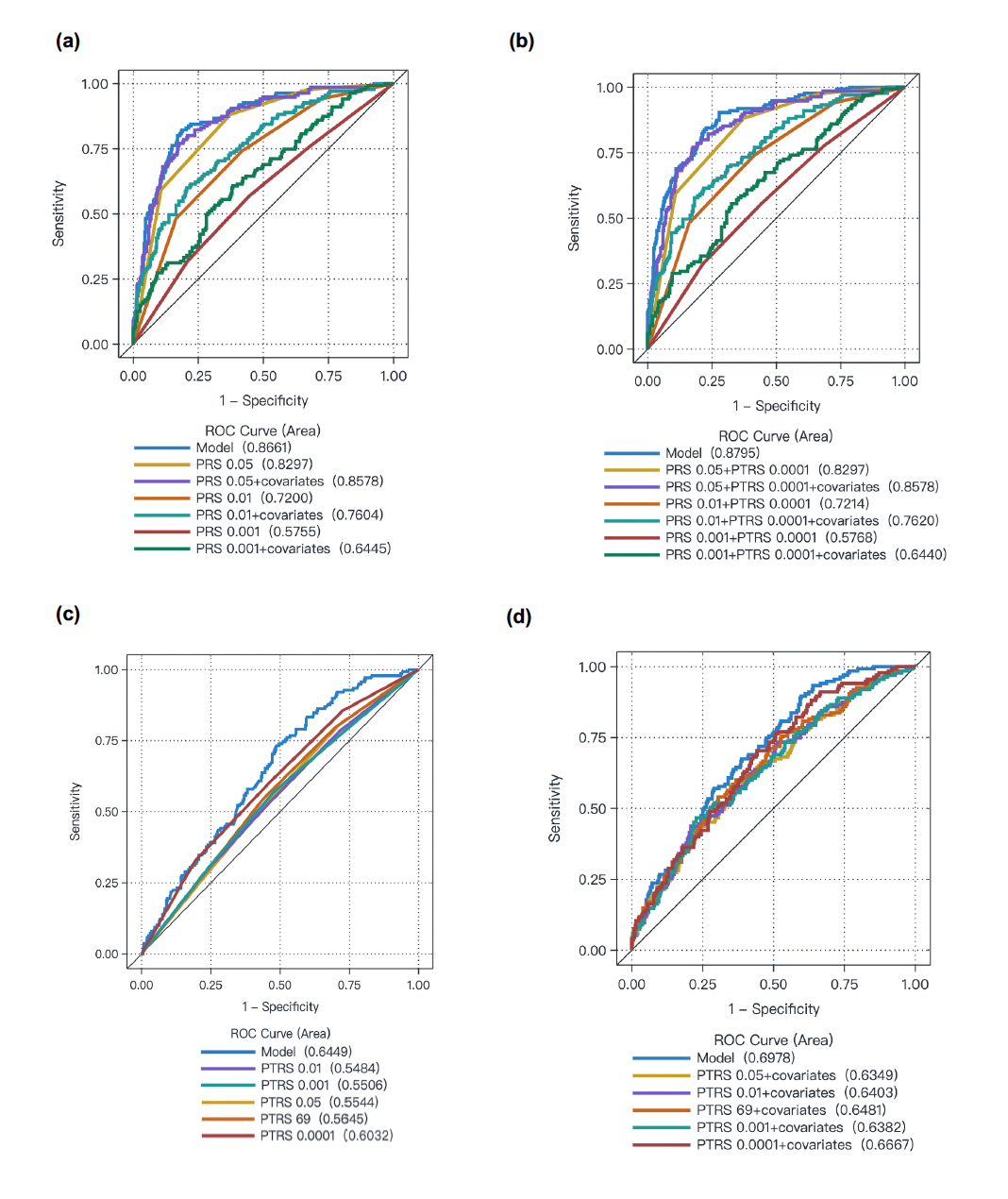
**

**Supplementary Figure 8. The ROCs and AUCs of SSBP risk evaluations models with PRS (a), PRS and PTRS combinations (b), PTRS with no covariates (c) and PTRS with covariates (d) in validation dataset.**
